# Signatures of selection underpinning rapid coral adaptation to the world’s warmest reefs

**DOI:** 10.1126/sciadv.abl7287

**Published:** 2022-01-12

**Authors:** Edward G. Smith, Khaled M. Hazzouri, Jae Young Choi, Patrice Delaney, Mohammed Al-Kharafi, Emily J. Howells, Manuel Aranda, John A. Burt

**Affiliations:** 1 Department of Biological Sciences, The University of North Carolina at Charlotte, Charlotte, NC, USA.; 2 Water Research Center & Center for Genomics and Systems Biology, New York University Abu Dhabi, Abu Dhabi, UAE.; 3 Khalifa Center for Genetic Engineering and Biotechnology, United Arab Emirates University, Abu Dhabi, UAE.; 4 Center for Genomics and Systems Biology, New York University, New York, NY, USA.; 5 Department of Fisheries Resource Development, Public Authority of Agriculture and Fisheries Resources, Kuwait City, Kuwait.; 6 National Marine Science Centre, Faculty of Science and Engineering, Southern Cross University, Coffs Harbour, NSW, Australia.; 7 King Abdullah University of Science and Technology, Thuwal, Saudi Arabia.

## Abstract

Coral populations in the world’s warmest reefs, the Persian/Arabian Gulf (PAG), represent an ideal model system to understand the evolutionary response of coral populations to past and present environmental change and to identify genomic loci that contribute to elevated thermal tolerance. Here, we use population genomics of the brain coral *Platygyra daedalea* to show that corals in the PAG represent a distinct subpopulation that was established during the Holocene marine transgression, and identify selective sweeps in their genomes associated with thermal adaptation. We demonstrate the presence of positive and disruptive selection and provide evidence for selection of differentially methylated haplotypes. While demographic analyses suggest limited potential for genetic rescue of neighboring Indian Ocean reefs, the presence of putative targets of selection in corals outside of the PAG offers hope that loci associated with thermal tolerance may be present in the standing genetic variation.

## Teaser

Population genomics reveals loci associated with coral adaptation to thermally extreme reefs.

## INTRODUCTION

Climate change will have profound impacts on the marine biosphere, across all levels of organization from genes to ecosystems (*1*, *2*). One of the most vulnerable ecosystems is coral reefs that are estimated to be home to over a quarter of all marine species (*3*). Reef-building corals have suffered extensive declines in recent decades, most notably because of widespread mass bleaching events in response to temperature extremes (*4*, *5*). Corals’ sensitivity to thermal stress, their long generation times, and sessile lifestyle have led to suggestions that corals may not keep pace with the forecasted increases in sea surface temperatures (*6*). Nevertheless, corals’ presence in acutely stressful thermal environments (*7*–*10*), such as highly variable tide pools where temperatures can exceed 34°C over the summer tidal cycle (*7*), suggests that the capacity to cope with elevated temperatures exists in some coral populations. The ability to survive in these highly variable environments is attributed to frontloading of stress-associated genes (*11*), multilocus adaptation (*12*), association with thermally tolerant strains of Symbiodiniaceae (*13*), and distinct microbial communities (*14*). While some corals have adapted to environments experiencing acute temperature events (time scale: hours), coral adaptation to environments exposed to prolonged high temperatures (time scale: weeks to months) is comparatively understudied. Understanding the processes and mechanisms driving coral thermal adaptation in these environments will provide a deeper understanding of their adaptive capacities and improve forecasts of coral responses to climate change.

Southern Persian/Arabian Gulf (PAG) reefs represent an ideal location to study coral responses to prolonged exposure to high temperatures that already exceed forecasts for reefs elsewhere in this century. These reefs experience the highest temperatures known for coral reefs, where summer temperatures can exceed 37°C and remain consistently above 34°C for over 1 month (*15*–*17*). These corals are adapted to this thermal regime, outperforming conspecifics at elevated temperatures and exhibiting the highest known bleaching thresholds globally (*15*, *18*, *19*). While southern PAG corals predominantly associate with a stress-tolerant algal symbiont, *Cladocopium thermophilum* (formerly *Symbiodinium thermophilum*) (*20*–*24*), thermal stress assays performed on aposymbiotic coral larvae demonstrate higher survival at elevated temperatures (*18*) and have identified a heritable contribution of host genotype (*25*) and epigenotype (*26*) to thermal tolerance. Notably, coral adaptation to the extreme conditions of the PAG must have been rapid considering the young age of this sea with modern coastlines established only ~6000 years ago following the Holocene transgression (*27*). While there is evidence to suggest that some corals within the southern PAG represent a distinct subpopulation of their species (*18*, *23*), the evolutionary history of coral populations in this young sea and patterns of connectivity across the PAG have not been characterized yet. Furthermore, little is known about the genomic targets of selection that enable symbiotic corals to survive the extreme PAG conditions relative to their counterparts in the neighboring Gulf of Oman (GO), which experience more benign thermal conditions.

The adaptation of southern PAG corals to extreme temperature makes them an invaluable ecological resource in light of the recent thermally-induced coral mortality events that have spanned the tropics (*4*, *28*). These events have occurred at temperatures that PAG genotypes experience for multiple months annually, and, consequently, it is essential to understand their evolutionary origin, distribution, and potential for genetic rescue of threatened reefs elsewhere. Furthermore, identifying targets of positive selection in the PAG will help elucidate the processes underpinning coral thermal tolerance. To this end, we characterized the demographic history, genetic connectivity, and targets of selection in eastern Arabian populations of the widely distributed pantropical brain coral, *Platygyra daedalea*, using a restriction site–associated DNA sequencing (RADseq) approach.

## RESULTS AND DISCUSSION

### Dataset

**Fig. 1.**
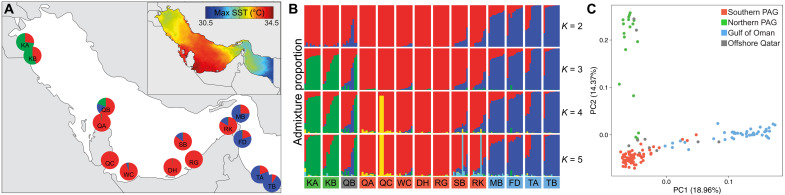
Population structure of coral populations along the eastern Arabian Peninsula. STRUCTURE analyses indicating admixture proportions averaged across populations [pie charts in (**A**)] and for each individual [bar charts in (**B**)]. Inset: Annual maximum mean sea surface temperature (SST) from 2004 to 2014 (*23*). (**C**) Principal component analysis (PCA) plot showing segregation of individuals based on allele frequencies. Each point is colored according to the sample’s collection location (blue, GO; green, northern PAG; gray, offshore Qatar; red, southern PAG).

We selected the brain coral *P. daedalea* as our model as it is widely distributed and an abundant and hardy member of PAG reefs (*29*, *30*). Furthermore, this species is an emerging model for coral studies, with numerous genomic resources available (*18*, *25*, *26*). We generated double-digest RAD (ddRAD) sequencing libraries from 168 *P. daedalea* colonies from 15 populations spread across the entire length of the PAG and the neighboring GO (table S1). The sampled reefs span a coastline of >1700 km and included reefs on either side of the narrow (50 km) Strait of Hormuz, which has been proposed as a putative barrier to gene flow between the PAG and GO (*31*). Furthermore, within the PAG, we also sampled from offshore and northern reefs, which could act as regional refugia as they experience cooler maximum temperatures than the better-studied inshore southern PAG locations (*32*). Our sampling sites spanned a range of thermal regimes with maximum monthly mean temperatures ranging from 34.4°C in the southern PAG to 31.6°C in the GO (*23*). The ddRAD libraries for these analyses were prepared with the enzymes Ase I and Bst BI, and after filtering, alignment, and variant calling, we identified 4963 single-nucleotide polymorphisms (SNPs) from 146 individuals in 14 populations (Fig. 1A). The median genotype sample depth was 85X.

### Regional structuring in present-day coral populations

While PAG corals’ bleaching thresholds are higher than reefs elsewhere (*15*), anomalous thermal events can occur in the PAG and have caused declines in the coral populations in recent decades (*17*, *29*). These events, coupled with the decimation of reefs associated with local anthropogenic activities, render the PAG coral populations vulnerable (*33*). Therefore, connectivity between reefs is increasingly important to assist with reef recovery. Biophysical models suggest that PAG reefs will become more isolated under climate change (*34*); however, the population structure and connectivity of present-day reefs are not well characterized from an empirical standpoint. To identify population structure, we performed clustering analysis (Fig. 1, A and B) and principal component analysis (PCA) (Fig. 1C) on our Ase I–Bst BI ddRAD dataset. We evaluated the presence of up to 10 populations within our dataset using STRUCTURE (*35*). At *K* = 2, the two clusters separate the samples into subpopulations corresponding to their location, with individuals from the PAG predominantly assigned to one subpopulation, and samples from the GO to the other. These population assignments correspond with the known symbiont genotypes in the region as PAG *P. daedalea* host *C. thermophilum*, whereas the Strait of Hormuz and GO individuals host symbionts belonging to the *Durusdinium* genus (*18*, *20*, *23*). The divide between the southern PAG reefs and those in the northern PAG is observed in clustering analysis at *K* = 3, with Kuwaiti corals assigned to a separate subpopulation. It is interesting to note that the admixture with the northern PAG cluster is largely restricted to the offshore Qatar site (QB) that sits at the interface between the northern and southern PAG populations. At the higher *K* values of *K* = 4 and *K* = 5, the assignments to new population clusters affect only a small subset of our samples in southern Qatar and in the more benign southern PAG sites, respectively. PCAs support our STRUCTURE results, with the first two principal components (PC) mirroring the patterns observed at *K* = 3; samples from the PAG and GO separate along PC1 of the PCA, while PC2 separates the individuals in the northern PAG reefs, from those in the south.

*P. daedalea* at PAG offshore reefs and reefs close to the Strait of Hormuz (QB, SB, RK) are more admixed with the GO population (proportion contributed by GO ancestry in individuals at *K* = 3: mean: 16%, max: 65%; Fig. 1B) than their inshore southern PAG counterparts (mean: 1%, max: 10%). The differences between inshore reefs, which experience higher thermal maxima, and offshore reefs are best exemplified by the different admixture proportions observed in northern Qatar. Despite the two sites (QA and QB) being situated 36 km apart, the mean admixture at *K* = 3 with non–southern PAG subpopulations at the offshore site (QB) is 37% compared with the inshore site (QA) at 2%, a pattern that is also reflected in the coral community diversity (*30*). The admixture results across the southern PAG are consistent with the results of three-population tests (table S2) but inconsistent with biophysical models of larval dispersal (*34*). The PAG current systems that supply offshore reefs with coral larvae should also feed proximal inshore sites, and therefore, low admixture proportions indicate that the more thermally extreme inshore reefs may select against migrants from the GO or cooler Iranian reefs. This observation is suggestive of isolation by environment (*36*) or phenotype-environment mismatch (*37*) and is supported by the results of an Akaike information criterion–evaluated modeling approach (*38*), which identified the optimal model as including both environmental and geographic distance as parameters shaping population genetic differentiation (fig. S1 and table S3). Given these constraints, the recent losses of locally adapted reefs across the southern PAG due to anthropogenic activities (*39*) will limit the capacity for recovery of inshore PAG populations from future stress-induced mortality events.

### Colonization of the PAG echoes the marine transgression after the last glacial maximum

**Fig. 2.**
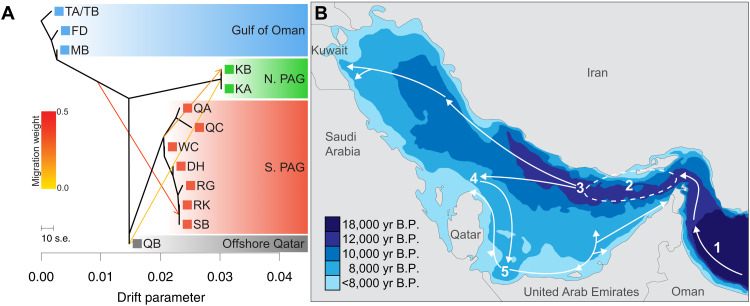
Past demography and migration among eastern Arabian coral populations. (**A**) TreeMix-inferred population tree with three migration events. Migration events are indicated by arrows and colored according to the migration weight. For details of site locations, see Fig. 1. (**B**) Schematic of PAG colonization by *P. daedalea* based on the TreeMix model. Colored contours show the shoreline at various points during the Holocene transgression after the last glacial maximum (*27*). White arrows signify inferred colonization routes. Dashed line highlights the putative location of the ancestral PAG population. Numbers indicate the relative order of events.

Understanding the processes that gave rise to present-day thermally adapted PAG populations will provide invaluable insight into the patterns and processes that drive local adaptation in coral populations, aiding assessments of corals’ potential to adapt to climate change. As the PAG is a young sea, it is likely that adaptation to these extreme conditions was rapid. To date, it is not known whether PAG corals quickly exploited the emergence of new habitat within the PAG after the marine transgression or if they arose from a subsequent, more recent, introduction. We set out to investigate past demography of PAG coral populations to characterize their colonization history and to identify migration routes between populations using TreeMix (*40*). TreeMix uses genome-wide allele frequency data to infer patterns of past population splits and mixtures between populations. The tree without any inferred migration routes explained 96.7% of the variance in relatedness between populations in our dataset. As the model underperformed for specific sites, with the largest residuals present in offshore and northern PAG sites (fig. S2), we sequentially added migration routes to the tree. We applied conservative filtering criteria (see Materials and Methods) to the migration-based trees to avoid overfitting and identified that the tree with three inferred migration routes provided the optimal fit. The three-migration route tree explains 98.7% of the variance and separates the coral populations into three major clades belonging to the GO (and Strait of Hormuz), the northern PAG, and the southern PAG (Fig. 2A), corresponding to our STRUCTURE (*K* = 3) and PCA analyses.

**Fig. 3.**
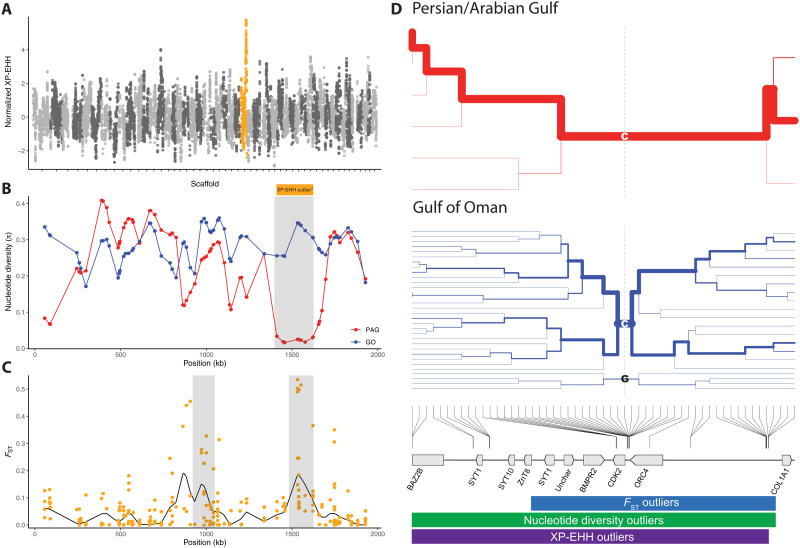
Candidate selective sweep in PAG *P. daedalea* populations. (**A**) Manhattan plot of XP-EHH scores across the longest 50 scaffolds. Scaffold 27 positions are shown in orange. (**B**) Variant-site nucleotide diversity across scaffold 27 for PAG and GO corals. Gray bar indicates outlier region (*Z* score > 5.0) where nucleotide diversity is reduced in PAG corals. Orange bar highlights XP-EHH outlier region (fig. S7). (**C**) *F*_ST_ tracks showing population differentiation between PAG and GO populations. Black line and orange points indicate Gaussian smoothed and individual SNP *F*_ST_ values, respectively. Gray bars highlight outlier regions (*P* < 10^−5^). (**D**) Haplotype bifurcation plots for PAG and GO haplotypes for scaffold 27 positions 1,411,154 to 1,621,714. Colors of each plot reflect the location of sampling. Labels indicate nucleotides at central position. Genomic position of SNPs, genes, and outlier locations is shown below bifurcation plots.

**Fig. 4.**
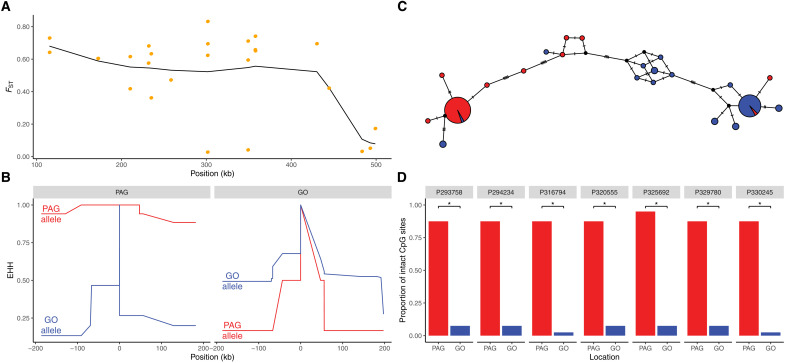
Candidate region under divergent selection between the PAG and GO. (**A**) Point (orange points) and window smoothed (black line) *F*_ST_ values across scaffold 441. (**B**) EHH in PAG (left) and GO (right) corals. Red lines denote the dominant allele in the PAG population, while blue lines indicate the dominant allele in the GO population. (**C**) Haplotype network encompassing all SNPs within the *F*_ST_ outlier region. Relative haplotype abundance is given by the diameter of the circles, and colored pie charts reflect the relative proportions of haplotypes from each population (red, PAG; blue, GO). (**D**) Proportion of intact CpG sites at highly differently methylated positions in introns of the gene XRN1 (5’-3’ exoribonuclease) (red, PAG; blue, GO).

Our TreeMix analyses of the demographic history of regional coral populations are consistent with reconstructed sea-level data after the last glacial maximum (Fig. 2, A and B). Coral populations migrated from the Indian Ocean northward along the coast of the GO and into the Strait of Hormuz (Fig. 2B-1). From the model, we can infer the presence of an ancestral PAG population that is likely to have been established along the Iranian coastline because of the initial infiltration to the south of Iran between 12,000 and 10,000 years before the present (yr B.P.) (Fig. 2B-2). There was a subsequent split into populations establishing reefs to the north, and in the southern/central PAG (Fig. 2B-3). After this split, populations were established at locations in the southern/central PAG consistent with the expansion of available habitats ~8000 years ago (Fig. 2, B-4 and B-5). In the southern PAG, new subpopulations were formed in a stepwise manner away from the pioneer population, after the establishment of the PAG’s modern coastlines ~6000 years ago (Fig. 2B-5). As the pattern of coral population establishment is consistent with marine transgression rather than the dominant surface currents that characterize this region (*27*, *41*), this supports early colonization of new habitats as the PAG formed during the Holocene transgression. The timing of this establishment in the PAG is corroborated by stairway plot analyses (*42*) of southern PAG coral populations, which show a strong bottleneck in the demographic history occurring between 20,000 and 30,000 yr. B.P., with declines in effective population size continuing up until 4000 yr. B.P. (fig. S3).

Migration routes inferred by TreeMix support migration into the PAG from the GO/Strait of Hormuz presumably through Iranian reefs but only to the site [Ras al Khaimah (RK)] closest to the Strait of Hormuz and the offshore island of Sir Bu Nair (SB). Meanwhile, we found no support for migration out of the PAG, even in models with up to 10 migration routes (fig. S2). Errors in the direction of TreeMix migration edges are possible (*35*, *43*); however, the direction is consistent across different inferred migration edges and is corroborated by three-population tests that show greater support for GO admixture in PAG populations (i.e., where individual tests take the form *PAG[PAG,GO]*: min. *Z* score = −21.7 *RK[SB,MB] P* = 5 × 10^−105^) compared with PAG admixture in GO populations (i.e., where individual tests take the form GO[PAG:GO] – min. *Z* score = −1.8 *MB[DH,TAB] P* = 0.23) (table S2). While our TreeMix and three-population test results do not conclusively rule out any gene flow between the two seas, it suggests selection in the GO against migrants from the PAG or the presence of a physical constraint. The unidirectional migration patterns across the Strait of Hormuz are consistent with the local oceanography as the Strait of Hormuz surface currents (which would transport coral larvae) flow from the GO into the PAG as a result of net water loss due to high surface evaporation rates within the Gulf (*41*). However, it is in contrast to demographic analyses for the sea urchin *Echinometra* sp. EZ, which has bidirectional migration across the Strait of Hormuz (*44*). It has been proposed that corals from warmer reefs, such as those in the southern PAG, may help accelerate adaptation of neighboring reef provinces through the supply of thermally tolerant genotypes (*45*). Successful genetic rescue in this context depends on thermal tolerance being heritable, which has been documented for coral larvae (*25*, *46*), and sufficient connectivity between thermally distinct reefs (*47*, *48*). This process of genetic rescue appears possible in other corals reef systems (*49*). However, the unidirectional migration patterns observed here suggest that there is historically limited export of PAG larvae, thereby reducing the capacity of PAG corals for genetic rescue of threatened Indian Ocean reefs.

### Selective sweeps in PAG corals

The exceptional thermal tolerance of PAG corals relative to their conspecifics elsewhere (*18*, *19*) is likely to have undergone positive selection, and, consequently, there should be signatures of selection at loci underpinning this trait within the genome. To identify candidate regions under positive selection, we sequenced a subset of samples from the southern PAG and GO with a more frequent cutting enzyme combination (Ase I–Msp I) to achieve higher sampling across the genome. We obtained 70,962 SNPs at a mean depth of 21X for 40 individuals. We applied three tests for selection on this SNP dataset that target different characteristics of selective sweeps: (i) *F*_ST_ outlier approach —testing for differences in allele frequencies between populations; (ii) population-specific loss of nucleotide diversity—identification of genomic regions where nucleotide diversity is reduced in a single population; and (iii) cross-population extended haplotype homozygosity (XP-EHH)—testing for differences in linkage disequilibrium (LD) that are specific to a single population (see Materials and Methods). Using this multiple outlier testing approach, with conservative filtering criteria, we identified a region in scaffold 27 containing SNPs that were identified as outliers in all three approaches (1,481,656 to 1,621,714; Fig. 3, A to C, figs. S4 to S8, and table S4). In this region, variant-site nucleotide diversity was reduced by 96%, haplotypes were longer (normalized XP-EHH scores up to 5.8), and Gaussian-window smoothed *F*_ST_ reached 0.19 (0.51 for individual SNPs). This region contains an SNP that is associated with larval survival under elevated temperature (fig. S9 and table S5) (*50*) and genes previously associated with coral bleaching responses and elevated thermal tolerance (*51*–*55*). Within this region, there are five genes, of which, synaptotagmin and cyclin dependent kinase 2 (CDK2) are noteworthy candidates for further investigation as they are differentially expressed by corals in response to thermal stress. Synaptotagmins are differentially expressed in response to thermal (*52*, *54*) and light stress (*51*), and SNPs associated with synaptotagmin transcripts have been shown to significantly correlate with survival at elevated temperatures in larvae of southern PAG corals (*25*). These genes play an important role in calcium homeostasis, a key component of the coral stress response (*11*, *52*, *54*, *56*), and potentially mediate exocytosis of the symbionts during bleaching (*57*). Meanwhile, CDK2 is a cell cycle gene that is highly differentially expressed between bleaching-sensitive and bleaching-resistant corals, with undetectable expression levels in the unbleached corals (*53*). Reduced expression of CDK2 is linked to a diapause state in coral larvae, and the capacity to implement this diapause-like state has been proposed to be associated with elevated thermal tolerance (*55*). On the basis of our current understanding of the function of these genes in corals, it is not possible to conclusively determine whether the selective sweep relates to the specific *P. daedalea*–*C. thermophilum* association found in the PAG or whether selection is acting independently on the *P. daedalea* host. However, emerging symbiosis model systems and recent advances in CRISPR-Cas9–based genome editing tools for corals offer new avenues to address this question (*58*, *59*).

The signature of selection on scaffold 27 is that of a strong selective sweep occurring in the PAG. While multiple haplotypes exist in the GO populations, a single haplotype dominates PAG populations (80% of haplotypes) in the combined outlier region (Fig. 3D). Furthermore, between positions 1,538,438 and 1,620,655 (28 SNPs), there is a single haplotype in the PAG population in our dataset. More frequent genomic sampling and sequencing of additional individuals may identify further haplotypes in the PAG population; however, the comparable decay in the GO population and the genomic range covered (compared with LD; fig. S10) provide strong support for a selective sweep in the PAG population. The low abundance of the dominant PAG haplotype in the GO samples may indicate that selection in the PAG acted on local standing genetic variation. However, we cannot rule out the export of PAG migrants despite the asymmetric gene flow seen in TreeMix analyses above, and further experimental analyses would be needed to test whether other environmental variables may be responsible for the sweep. Nevertheless, the presence of the putatively beneficial haplotype outside of the PAG is a promising indicator that the genomic basis for thermal tolerance exists outside of the PAG, giving hope for corals elsewhere to respond to future climate change if the genetic diversity of existing populations can be maintained.

### Selection for differentially methylated haplotypes

Methods to detect selective sweeps are likely to miss selection in regions where the signature is either weaker than or different to a classic hard sweep. For example, the largest *F*_ST_ values between the PAG and GO that we observed in our dataset were found in a region showing evidence of disruptive selection. Sixteen of the top 20 genome-wide Gaussian-smoothed *F*_ST_ values are found in a region spanning ~300 kb along scaffold 441, with *F*_ST_ values reaching 0.68 (Fig. 4A). Maintenance of the haplotypes is evident in both populations but for alternative haplotypes (Fig. 4B). The PAG and GO populations are primarily dominated by single haplotypes in our data (78% PAG; 55% GO), with these haplotypes differing at 21 of 23 SNP positions dispersed across ~300 kb (Fig. 4C). While reduced representation sequencing cannot give the complete picture of haplotype structure in this region, analyses with more dense SNP markers do support the high differentiation and extended LD observed at this locus (fig. S11). Within the selected region, there are two annotated genes, of which, 5’-3’ exoribonuclease (XRN1) has been shown to be significantly differentially methylated between the PAG and GO (see (*26*) and Supplementary Results). In corals, differential methylation has been shown to correlate with fitness under altered environmental conditions, and methylated genes show reduced spurious transcription and transcriptional noise (*26*, *60*, *61*). The plant XRN1 homolog, XRN4, has been shown to play a key role in thermal tolerance with the performance of knockout mutants strongly affected by the severity and duration of thermal stress (*62*, *63*). Improved survival of XRN4 knockout mutants under severe short-term thermal stress was attributed to the accumulation of stress response transcripts (*62*). This higher level of expression of heat response genes under nonstressful conditions mirrors the frontloading observed in thermally tolerant corals from acute thermal environments (*11*).

A study conducted by Liew *et al*. (*26*) identified differences in methylation at XRN1 based on comparisons of adult and sperm samples taken from PAG and GO coral populations. However, these analyses excluded sites that were identified as containing SNPs in any of the samples. As genotype can strongly influence methylation (*26*, *61*), particularly through the provision of CG dinucleotides, we reanalyzed the data of Liew *et al*. (*26*) without the SNP filter to assess whether selection may act to maintain high XRN1 methylation in the PAG population. Methylation at each position in XRN1 is correlated between PAG and GO samples (*r*^2^ = 0.74), but there are notable outliers where mean methylation across individuals is >4-fold lower in the GO (fig. S12). These outlier positions showing evidence of mutable CpG sites may be important for larval thermal tolerance as their methylation levels correlate more strongly with larval heat survival than the rest of the positions (figs. 12 and 13 and table S6). We sequenced a subset of these sites (seven methylated CpG sites in four introns) in our samples to test whether the lack of methylation in GO samples was due to the absence of a CpG site. We found significant differences in the presence of the intact CpG site between the two populations, with the CpG sites present in >87% of PAG haplotypes but <8% in GO haplotypes (Fisher’s exact tests, *P* < 0.05; table S7).

While elevated evolutionary rates associated with heavily methylated genomic regions may explain the substantial divergence between the dominant PAG and GO haplotypes, it does not exclusively explain the low genetic and haplotypic diversity that we observe. As methylated cytosines are 10 times more likely to transition to thymine (*64*), under neutral processes, it would be expected that the more methylated samples (i.e., PAG corals) would have more losses of CpG sites because of the higher mutation rates. We find the opposite whereby the loss/absence of the CpG sites is consistently greater in the GO across multiple loci located in multiple introns. The phenotypic effect of the increased intronic methylation resulting from differences in CpG availability is unclear but may involve changes in transcription (*65*–*67*). Nevertheless, considering that the presence/absence of CpG sites does not appear to be stochastic or associated with deamination of methylated cytosines, we propose that selection is acting in this region to preserve methylation substrate within PAG populations.

Together, our results show that *P. daedalea* in the PAG represents a young coral population that rapidly adapted after the Holocene marine transgression. We highlight the role of positive and disruptive selection in coral adaptation across an extreme thermal gradient and provide evidence for evolutionary interplay between the genome and epigenome. Considering the unprecedented pace of recent climate change, genetic rescue of vulnerable Indian Ocean populations by PAG corals may be limited by insufficient gene flow. Nevertheless, the presence of putatively beneficial haplotypes in the GO suggests that thermal tolerance genotypes may exist in the standing genetic variation and emphasizes the importance of conserving coral reef genetic diversity.

## MATERIALS AND METHODS

### Sample collection, processing, and DNA extraction

Samples of *P. daedalea* were collected from 15 reefs along the northeastern Arabian Peninsula, sampling at sites in the northern and southern PAG, and the GO. At each site, small fragments (<1 cm^3^) were collected via SCUBA using a hammer and chisel, and each sample was placed in a separate Ziplock bag. Upon return to the boat, samples were frozen on dry ice or stored in ethanol or salt-saturated dimethyl sulfoxide (DMSO). Frozen samples were stored at −80°C, and ethanol/salt-saturated DMSO samples at 4°C, before DNA extraction. DNA extraction was performed using a modified SDS-based lysis, described elsewhere (*16*, *24*).

### ddRAD sequencing library preparation

The ddRAD library preparation followed the protocol described by Peterson *et al*. (*68*), with minor modifications. Briefly, restriction digests were performed in a single reaction that included both restriction enzymes (Ase I–Bst BI; Ase I–Msp I), Tango buffer (Thermo Fisher Scientific), and 500 ng of DNA. After incubation at 37°C for 8 hours, the reactions were cleaned using 1.5× AMPure beads. Digested DNA was quantified using the Quant-it High Sensitivity dsDNA kit according to the manufacturer’s recommendations. Normalized quantities of DNA were ligated to barcoded adapters and pooled before cleanup with 1.5× AMPure. Size selection [Ase I–Bst BI, 338 to 412 base pairs (bp); Ase I–Msp I, 350 to 500 bp] was performed in triplicate for each pool using a 2% agarose, dye-free Pippin Prep cassettes with internal standards. Amplification and addition of indices were performed on size-selected DNA, split across six reactions (table S8) and using the following polymerase chain reaction (PCR) conditions (one cycle of 98°C for 1 min; eight cycles of 98°C for 10 s, 62°C for 30 s, 72°C for 30 s; and one cycle of 72°C for 10 min). Libraries were quantified by quantitative PCR using the Kapa Illumina Quantification Kit and pooled in equimolar amounts before sequencing. Pooled libraries were sequenced on a HiSeq 2500, either as a single lane in full output mode or using the rapid run mode. Sequencing was performed with 40 to 50% PhiX.

### Read processing

The processing of reads for both Ase I–Bst BI and Ase I–Msp I datasets followed the approach of dDocent v2.2.25 (*69*) using the *P. daedalea* assembly (*26*) as the reference. Quality trimming and adapter removal of raw sequencing reads were performed in trimmomatic (v0.33). Demultiplexing of sequences and additional filtering were performed using the Stacks (v1.47) process_radtags program (*70*). Reads were aligned to the *P. daedalea* reference genome v1.0 using bwa mem (v0.7.17) using the default parameters. Bam files were subsequently filtered requiring that reads mapped with a minimum quality score of 30. The bam files were used to call variants in freebayes (v1.1.0.9). Variants were called in freebayes using default parameters on alignment files for both Ase I–Bst BI and Ase I–Msp I datasets. The resulting variant calls were filtered using the RAD-specific filtering approach recommended as part of the dDocent pipeline (*69*) (https://github.com/jpuritz/dDocent) with some modifications (table S9). Lastly, as linkage disequilibrium can affect inference of population structure and demography, we pruned our Ase I–Bst BI SNPs for markers in approximate linkage equilibrium in PLINK v1.90 (*71*) using with a window size of 10 kb, a step size of 5 kb, and an *r*^2^ threshold of 0.1.

### Population structure and demography

To resolve population splits and potential admixture between populations, we used the software TreeMix v1.13 (*40*). TreeMix calculates a maximum likelihood tree and subsequently adds migration events between populations to improve the fit between the model and the data. The input for TreeMix was created from the variant call format file using the populations module of Stacks (*70*). As there may be variation between TreeMix runs (*40*), we performed 30 iterations of TreeMix, each using a new random seed, for migration events from 0 to 10. The Muscat-based sites (TA and TB; see Fig. 1, table S1) were combined and chosen as the root population as they likely represent the oldest sites studied (*27*). The outputs of each run were visualized using the functions in the R script provided with TreeMix (plotting_funcs.R).

We assessed population structure among our samples using PCA and model-based clustering. PCA was performed in PLINK. The eigenvectors and eigenvalues were imported into R for plotting. We used the model-based clustering program STRUCTURE v2.3.4 (*35*) to assign population membership probabilities for each sample using the correlated allele frequencies model with admixture. Structure was run for *K* values between 1 and 10, and 20 iterations were performed using different random seeds. Structure was run for 100,000 Markov chain Monte Carlo repetitions (after burn-in of 100,000), and no prior population information was used in the model. To visualize the structure assignments, we used CLUMPP (v1.1.2) (*72*) to match cluster labels across runs and calculated the mean proportions across all replicates. All structure plots were generated in ggplot using the mean proportions calculated by CLUMPP.

### Outlier detection

To detect outliers in our Ase I–Msp I dataset, we performed three tests for genomic outliers based on different characteristics of selective sweeps.

#### F_ST_ outliers

We compared allele frequencies between the two populations (PAG and GO) using Wright’s fixation index. The calculation of *F*_ST_ was performed in the populations module of Stacks (*70*) using the analysis of molecular variance (AMOVA) approach. *F*_ST_ values were kernel smoothed using a Gaussian-weighted function centered on each SNP (*70*, *73*). The sigma value (25 kb) used in our analyses was smaller than the value used previously for three-spine stickleback to accommodate our more fragmented genome (N50 = 665 kb; assembly size = 843 Mb). Because of variation in the distribution and density of SNP-associated RAD sampling, the Gaussian smoothed *F*_ST_ values were bootstrap resampled with replacement for each SNP window to assess departure from a null distribution of genome-wide averages under the constraints of RAD sequencing. For computational efficiency, we used the approach of Hohenlohe and co-workers (*73*), sequentially increasing the number of bootstrap resampling replicates up to a maximum of 10 million replicates to improve accuracy for those windows at the tail of the distribution.

#### Nucleotide diversity

We compared the variant-site nucleotide diversity between the two populations to identify regions where loss of diversity is associated with a single population. Variant-site nucleotide diversity was calculated in Stacks populations module and Gaussian window averaged as described above. We used the *Z* score approach from Hazzouri and co-workers (*74*) to identify candidate selective sweeps

$$Z=\frac{x_{i}-\bar{x}}{s.d.(x)}$$

where *x_i_* is ${\text{log}}_{2}(\frac{\pi_{\text{PAG}}}{\pi_{\text{GO}}})$ in each genomic window, $\bar{x}$ is the mean of *x*, and s. d. (*x*) is the SD of *x*. Z scores in the lower tail of the distribution that were greater than 5 SDs from the mean were classified as outliers.

#### EHH and XP-EHH

To identify population-specific difference in LD, we used the XP-EHH metric (*75*) using phased genotype calls from Beagle 4.1 (*76*). As the calculation of EHH requires measuring the extent of LD from each SNP, it is of limited utility in short scaffolds. Consequently, we only calculated EHH (and XP-EHH) for scaffolds >300 kb and with >20 SNPs while recognizing that our power to detect outliers at the ends of scaffolds will be compromised. EHH and XP-EHH were calculated in selscan v1.1.0b (*77*) and normalized to account for genome-wide differences in haplotype length between the PAG and GO populations using the normalization script. SNP positions were identified as outliers if the absolute XP-EHH score was in the top 1% tail of the statistic (corresponding to an absolute XP-EHH ≥ 2.81511) and the XP-EHH score was positive, reflecting the presence of extended haplotypes in the PAG population. We acknowledge that there are limitations to using LD-based approaches with reduced representation datasets; however, a comparative approach should enable detection of differences between populations despite these limitations. Haplotype bifurcation plots were generated using rehh (*78*), and median-joining haplotype networks were generated in popart (*79*).

### Methylation

We designed primers to target strongly differentially methylated sites (4- to 12-fold greater methylation in the PAG; fig. S12) using the *P. daedalea* genome annotation. These positions were identified as intronic based on the genome annotation and verified through comparison to the XRN1 sequence from *Acropora digitifera* (accession no. XP_015751869) (*80*). The primer location within the introns was largely dependent on the amenability for primer design (i.e., product size of 300 to 1000 bp, 40 to 60% GC (guanine-cytosine) content, *T_m_* 56° to 58°C, and specificity of primer sites). We identified five primer pairs that successfully amplified seven differentially methylated sites across four XRN1 introns (table S10). We amplified these intronic loci from the 40 Ase I–Msp I samples using the high-fidelity enzyme PrimeStar GXL and the following conditions (1 cycle of 98°C for 10 s; 30 cycles of 98°C for 10 s, 58°C for 20 s, 68°C for 1 min; and 1 cycle of 68°C for 5 min). PCR amplicons were sequenced at Bioneer (Korea). Where slippage (typically caused by homopolymer A/T runs) resulted in ambiguous calls, we resequenced samples using internal primers. Chromatograms were manually inspected at differentially methylated sites, and the genotype was recorded for each sample. The distribution of intact CpG sites across the two populations was tested using Fisher’s exact tests, implement in R.

## Supplementary Materials

### This PDF file includes:

Supplementary Text Figs. S1 to S15 Tables S1 to S12 References

## Notes

View/request a protocol for this paper from *Bio-protocol*.
