# Supplementary material for: Signatures of selection underpinning rapid coral adaptation to the world’s warmest reefs
Source: Sci Adv. 2022 Jan 12;8(2):eabl7287. doi: 10.1126/sciadv.abl7287 (PMC10954036; doi:10.1126/sciadv.abl7287)
Supplement: Supplementary file 1 — Supplementary Text Figs. S1 to S15 Tables S1 to S12 References [file sciadv.abl7287_sm.pdf]

Science Advances
AAAS

Supplementary Materials for

Signatures of selection underpinning rapid coral adaptation to the world's warmest reefs

Edward G. Smith*, Khaled M. Hazzouri, Jae Young Choi, Patrice Delaney, Mohammed Al-Kharafi, Emily J. Howells, Manuel Aranda, John A. Burt

*Corresponding author. Email: esmit245@uncc.edu

Published 12 January 2022, *Sci. Adv.* **8**, eabl7287 (2022)
DOI: 10.1126/sciadv.abl7287

This PDF file includes:

Supplementary Text
Figs. S1 to S15
Tables S1 to S12
References

Supplementary Text

Supplementary Methods

Read processing and SNP filtering

Sequencing yielded a mean of 2.6M (min = 26,818; max = 8,223,542) and 3.0M (min = 1,176,860; max = 4,865,064) raw paired-end reads per individual for the Ase-BstBI and Ase-MspI libraries, respectively. After QC, alignment and variant calling, SNP filtering was performed using the RAD-based filters recommended in the dDocent pipeline (*69*) using the script dDocent_filters.sh. For steps requiring dataset-specific customization, we used the following parameters for our two datasets (Supplementary Table 9). As a result of filtering, a total of 12 individuals were removed from the AseI-BstBI dataset, including the removal of one site (MA) from our analysis as the number of samples at that site dropped below eight individuals after filtering for individuals with excessive missingness. No individuals were removed from the AseI-MspI dataset.

Analysis of changes in effective population size

We used Stairway plot v2 (*42*) to estimate historical changes in effective population size. Stairway plot estimates past demographic changes using a model-free approach, circumventing issues with identifying the correct demographic model and has successfully been used for coral populations (*49*). We used ANGSD v0.911-44 to generate a site frequency spectrum for this analysis. Briefly, ANGSD (*81*) calculated genotype likelihoods for southern PAG corals from sites with little to no admixture (Sites=QC,WC,DH,RG; n=45) from the BAM files generated by BWA-MEM (*82*) using conservative filters (minInd=40, setMinDepth=900, SNP_pval=1e-6, hwe_pval=0.01). The site frequency spectrum was subsequently estimated from the genotype likelihoods using the module realSFS. Estimating the effective population sizes and dating historic changes in the size of the population is dependent on the mutation rate and the average generation time of the organisms. We used a per generation mutation rate of 4.69×10^{-8}, based on inferred per year mutation rates for Porites corals (*83, 84*) and a generation time of 34 years (*85*). We plotted the output from Stairway plot in R using ggplot.

Correlation of genetic, geographic and environmental distances

We used an information theoretic approach to test factors that could be associated with the population structure observed among *P. daedalea* individuals based on the approach of Saenz-Agudelo and coworkers (*38*). We evaluated 7 models using a sample size adjusted Akaike Information Criterion. These models comprised of combinations of three potential factors presumed to potentially affect local genetic structure: geographic distance, environmental conditions, and the presence of a dispersal barrier at the Strait of Hormuz. Geographic distance was calculated as the minimum distance over water. As temperature and salinity conditions in the PAG are considered 'marginal' relative to reefs elsewhere (*86*), we calculated environmental distance based on the maximum and minimum temperatures and salinities obtained from MODIS-Aqua monthly averaged SST (4μm nighttime) and Copernicus Marine Environment Monitoring Service (CMEMS) GLOBAL_ANALYSIS_FORECAST_PHY_001_024 monthly-averaged salinity, respectively. We performed principal component analysis between sites using standarised environmental variables and environmental distance was subsequently calculated as the Euclidean distance between sites. The presence of a dispersal barrier was included in our model sets as it has

been proposed that the direction of surface currents at the Strait of Hormuz (i.e., into the PAG) limit the export of larvae from the PAG to the Gulf of Oman (*23, 31, 41*). The analyses were performed in R using the script “Information_theoretic_aproach_script.R”, adapted for our set of models.

Estimation of linkage disequilibrium

We estimated linkage disequilibrium for PAG and GO populations using vcftools v0.1.14 (*87*). LD was calculated as the squared correlation coefficient between genotypes (--geno-r2) for bi-allelic SNPs within 100kb of each other (--ld-window-bp 100000) with a minor allele frequency of 0.1 (--maf 0.1).

Reanalysis of CpG methylation in outlier regions

We reanalyzed the data of Liew and coworkers to assess the impact of removing potential SNP sites from methylation analyses. To do so, we re-filtered the data without the SNP filter (*88*) (https://github.com/lyijin/working_with_dna_meth) and compared the results for our outlier regions with the original study (*26*). For the genes in scaffold 27, we did not observe significant differences in methylation between PAG and GO corals regardless of whether the putative SNP filter was applied (Supplementary Table 11).

For Pdae15888 (XRN1) on scaffold 441, we documented putative mutated methylation sites in the dataset of Liew and coworkers (*26*) as positions where the methylation was 0% for both of the corresponding adult tissue and sperm samples from at least one individual. Subsequently, we compared the mean correlation of these positions with the larval heat survival index (*26*). We performed permutation tests in R to identify whether the mean Pearson’s correlation with heat survival index for putative mutated methylation positions was greater than positions without evidence of mutation (number of permutations = 10^7).

Three-population test

The three-population test was performed to test the robustness of inferred migration routes. The three-population test of the form $f_3(A;B,C)$ tests for tree-like behaviour between the three populations, with a significantly negative f_3 statistic indicating population A is admixed. We performed the three-population test for all population pairs and accounted for multiple testing using a Benjamini-Hochberg correction.

Identification of Scaffold 441 genotype structure

To visualize genotype structure along Scaffold 441 in the adult samples from the Liew et al. study (*26*), we generated genotype plots using Genotype Plot (https://github.com/JimWhiting91/genotype_plot). The SNPs were called in Bis-SNP v1.0.1 (https://github.com/lyijin/pdae_dna_meth/tree/master/genetic_contribution/bissnp) and were filtered for high confidence sites where the genotype of the adult sample matched the corresponding sperm sample.

Supplementary Results

Treemix

We performed 30 runs of Treemix for migration events between zero and ten. Eleven of the 30 runs, failed to calculate the likelihood for migration events above five (i.e., m > 5). As these runs were characterized by lower likelihoods compared to successful runs, it suggests failure to converge on suboptimal solutions and therefore these were excluded from further analyses (Supplementary Figure 14). The plot of the model likelihood reaches saturation at three migration events, with the addition of further migration events providing little improvement on the proportion of explained variance (Supplementary Figure 2). Consequently, we infer that the model with three migration events best explains our data. It is worthwhile to note that the largest residuals in our dataset are for populations SB and RK, suggesting that these populations are the least well explained by our Treemix model. This could be indicative of admixture with unsampled populations in the region, which considering their location, could be from Iranian reefs to the north. Resolving the complex history of these populations with additional sampling of offshore and Iranian reefs would provide an interesting target for further study.

Scaffold27 outlier region and larval survival at elevated temperatures

As the selective sweep on scaffold27 could be related to thermal tolerance, we analyzed data for this genomic region from a recent study (*50*) that performed experimental crosses using PAG and GO corals. Our goal was to ascertain whether there was an association between positions at this locus and survival of coral larvae at elevated temperatures. We extracted 13 SNPs across nine 2bRAD tags that were within the scaffold27 outlier region in at least one outlier test, and performed a linear regression between minor allele frequency (MAF) of the larval family (independent variable) and survival at elevated temperatures (dependent variable). There is a significant relationship between MAF and larval survival for one of these SNPs (Supplementary Table 5, Supplementary Figure 9). This SNP was also significantly correlated with larval survival in an independent genome-wide analysis involving a larger number of coral families (i.e., PAG, GO, and PAG×GO crosses) (*50*).

Methylation of XRN1

As methylation data are available for *P. daedalea* samples from the PAG and GO, we investigated whether the observed differentiation in XRN1 gene was reflected in methylation patterns. While a significant difference in methylation of this gene was observed using the filtering criteria of Liew and coworkers (*88*)(position is significantly methylated, median coverage of position > 10, methylation in all replicates of at least one treatment, with the addition of methylation must be present in both Gulfs; https://github.com/lyijin/working_with_dna_meth; Supplementary Figure 15, Supplementary Table 12), the effect size was reduced with the application of an additional filter removing methylated sites that were identified as a potential SNP in any individual (*26*). This suggested an effect of genotype on the epigenome at this locus. Therefore, we re-analysed the data keeping the putative SNP sites identified by Bismark in the analysis in order to identify sites strongly differentiated between water bodies to explore whether the epigenetic differences could be attributed to mutations causing loss/gain of methylation sites. While the majority of methylation positions were correlated between the two populations, there was a subset of positions that showed greater differences in methylation, predominantly with greater methylation in PAG samples (positions located below diagonal in Supplementary Figure 12). The inter-population methylation differences at these positions were primarily driven by individuals that show no methylation for both adult tissue and sperm samples; a pattern which could indicate the presence of a mutation impacting the methylation site. We decided to investigate these positions as they were significantly more correlated with larval survival under thermal stress than XRN1

positions unaffected by putative SNPs (Supplementary Table 6). We selected a subset of seven sites for further investigation of putative SNP sites using Sanger sequencing.

Sanger sequencing of highly differentially methylated sites in intronic regions of XRN1 revealed that the differences in methylation between the sites is driven by the presence/absence of the CG dinucleotide at the respective positions. The proportions of CG relative to non-CG alleles at the sites is significantly different between the PAG and Gulf of Oman for all sequenced locations (Supplementary Table 7). Furthermore, the consistency between allele frequencies across sites reflects the strong linkage disequilibrium in this region (Figure 3b).

As methylated cytosines are highly mutable, it would be expected that the differences in the presence/absence of the CG dinucleotides are a result of cytosine to thymine transitions. However, our data (Supplementary Table 7) do not support this expectation for the following three reasons. Firstly, we observe both transitions and transversions. Secondly, the XRN1 gene region in PAG populations is more methylated and therefore we would expect greater loss in this population. Lastly, although we do not know the ancestral state of these positions, we can postulate that the non-CG alleles may be ancestral as they are the most abundant alleles in the older populations

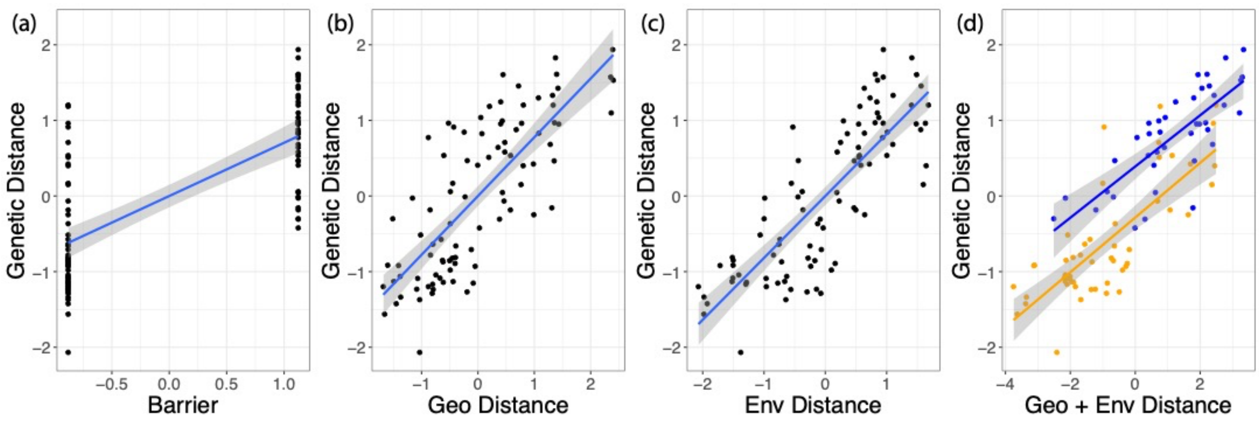

Fig. S1.

Correlation between pairwise genetic distance and geographic and environmental factors for *Platygyra daedalea* along the eastern Arabian Peninsula. Genetic distance is correlated with (a) the presence of a dispersal barrier at the Strait of Hormuz (low: sites within the same sea / high: sites across the Strait of Hormuz), (b) geographic distance, (c) environmental distance, and (d) model incorporating the presence of a barrier, geographic distance, and environmental distance. Orange points in (d) denote comparisons between sites on the same side of the Strait of Hormuz, blue points indicate comparisons between sites across the Strait of Hormuz.

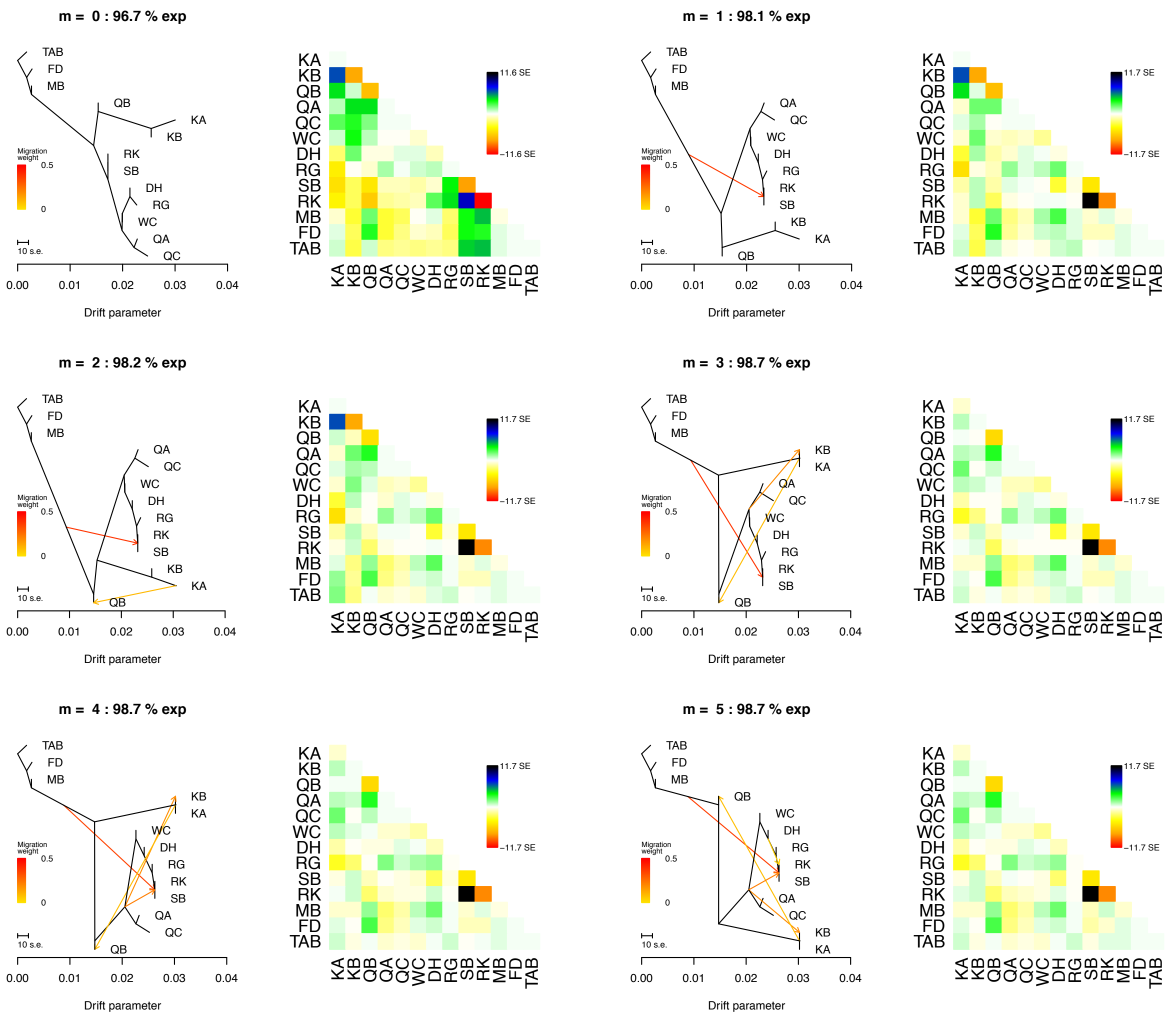

Fig. S2.

Treemix models and corresponding residual plots for migration events ranging from zero to five, with the highest likelihood at m=3.

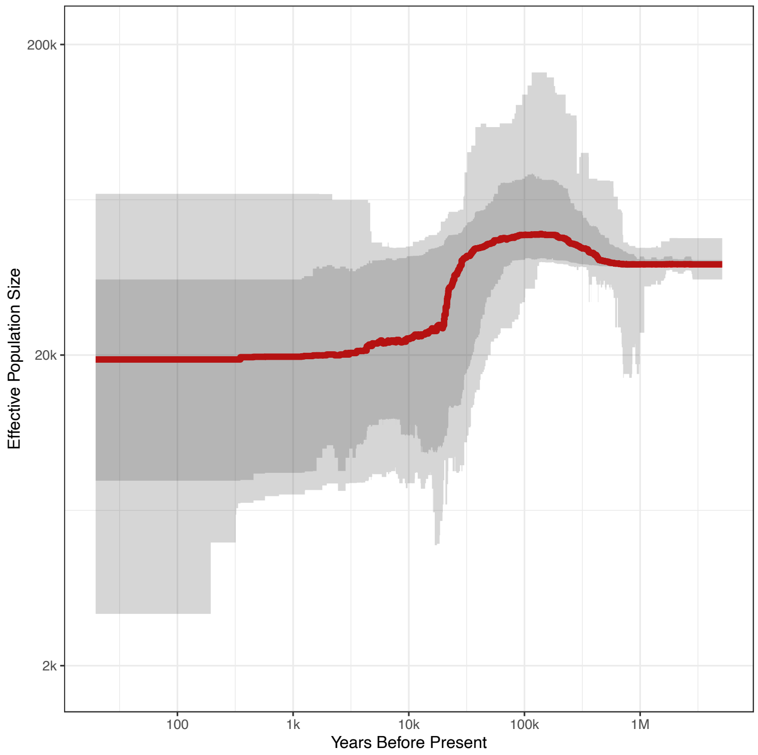

Fig. S3.
Stairway plot of historic changes in effective population size for southern PAG *P. daedalea*. The red line shows the median estimated effective population size with the 75% and 95% confidence intervals show in light and dark grey, respectively.

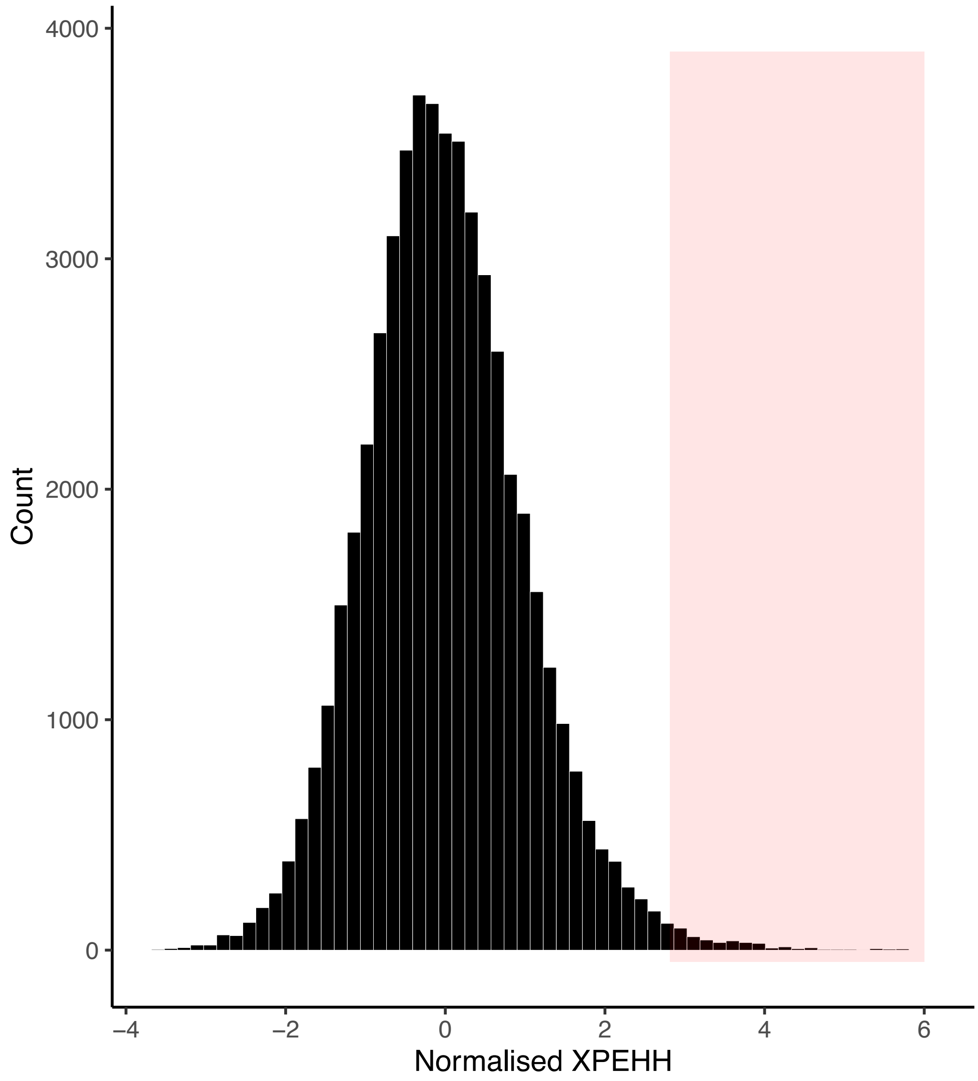

Fig. S4.
Histogram of normalized XPEHH values. Red shading highlights XPEHH scores exceeding the threshold and with the longer haplotypes present in the Persian/Arabian Gulf.

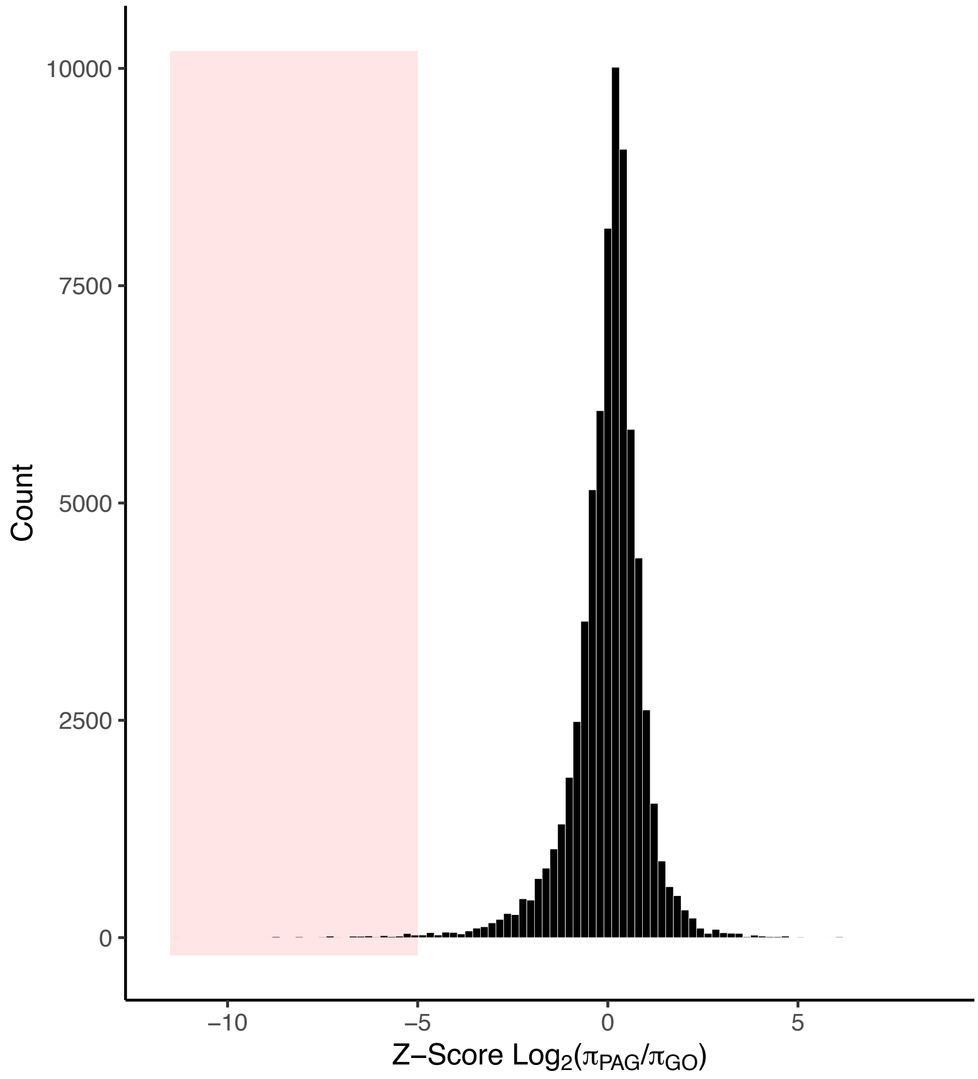

Fig. S5.
Distribution of Z-scores for $\log_2(\pi_{PAG}/\pi_{GO})$ in Gaussian-smoothed genomic windows. The lower tail of the distribution was used for the identification of outliers indicative of selective sweeps in the PAG population.

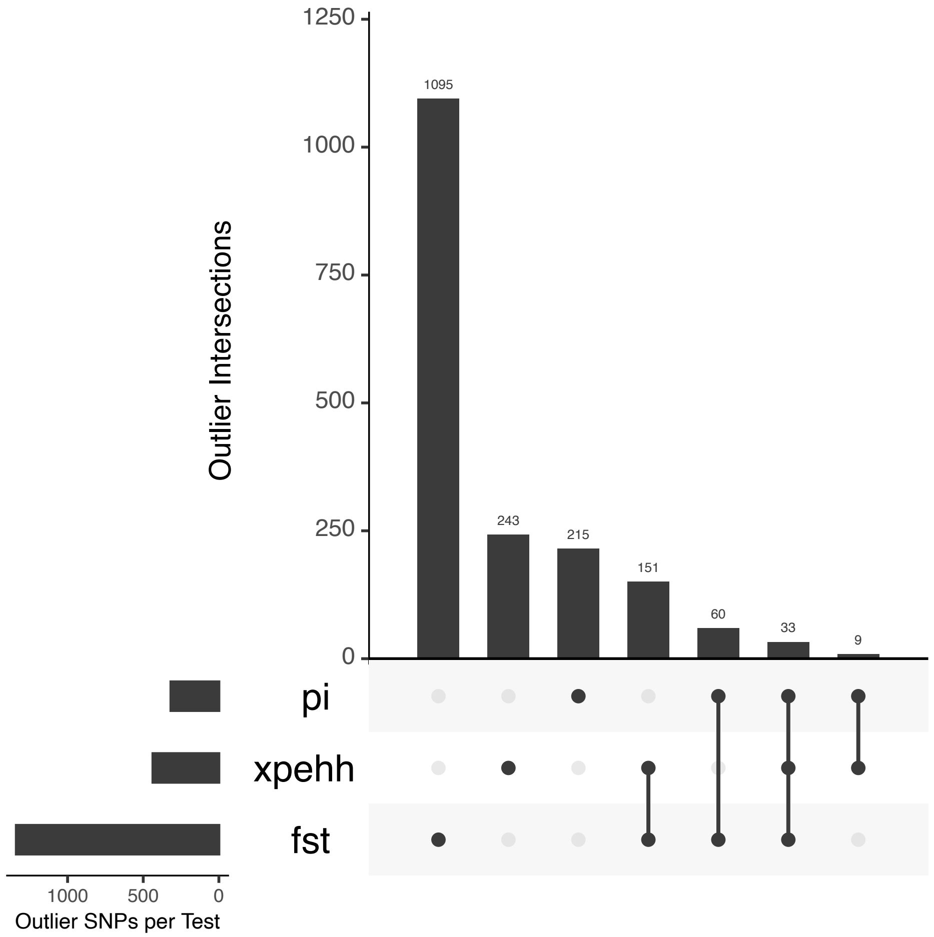

Fig. S6.
UpSet plot showing overlap of outlier SNPs identified between each method.

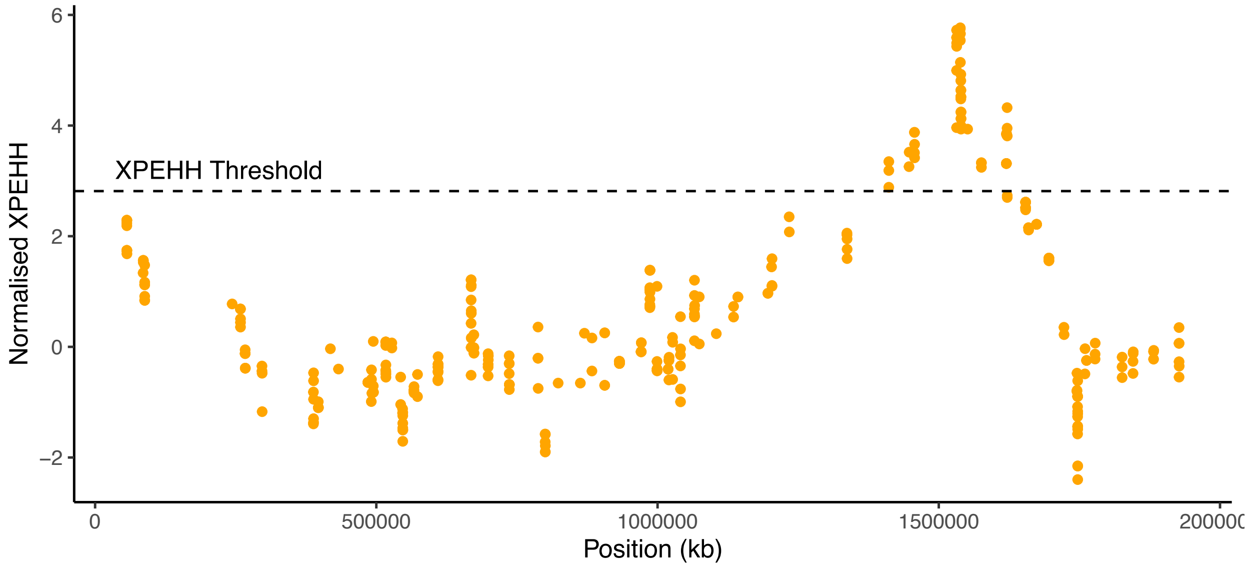

Fig. S7.
Normalised XPEHH scores across scaffold 27. Dashed line indicates the threshold above which positions were classified as outliers.

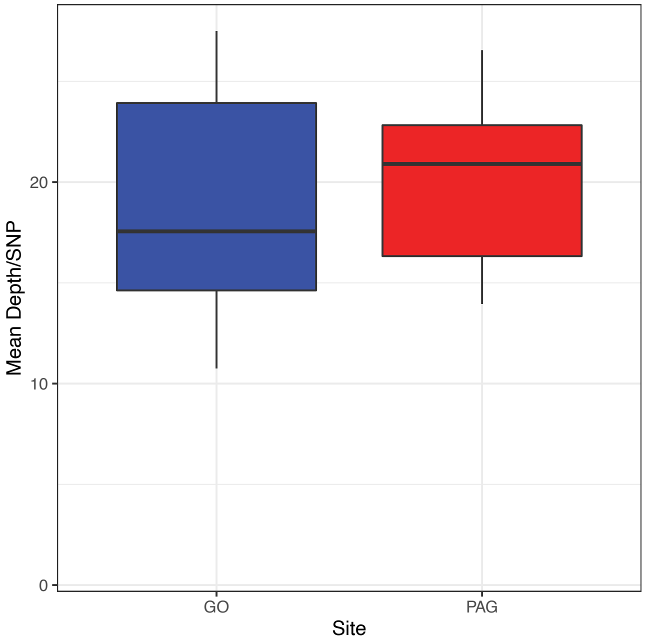

Fig. S8.

Box plot of mean depth for each SNP in the scaffold 27 outlier region shown in Figure 2 in the PAG and GO samples. As low coverage could result in signals of reduced nucleotide diversity and extended haplotype homozygosity seen in the PAG population, we checked whether read depth for this region was lower relative to the GO samples. We found a significant difference in mean read depth (paired t test, t=3.7713, df=54, p=0.0004046) with the higher depth found in the PAG samples, therefore the signatures of selection on scaffold 27 are unlikely to be a result of low coverage.

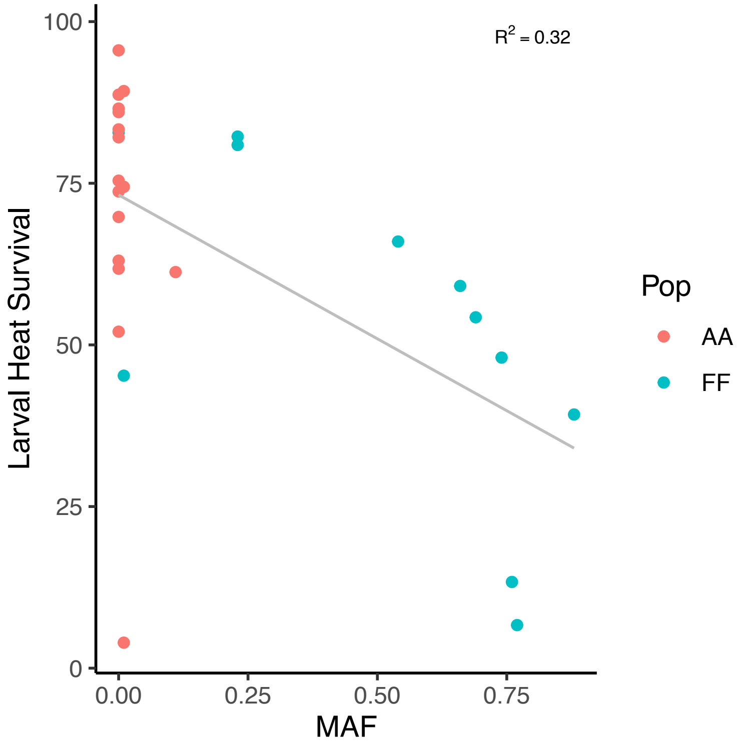

Fig. S9.
Regression plot for the SNP that has a significant relationship with larval heat survival. These data were extracted from the breeding and selection experiments in Howells et al. (*50*).

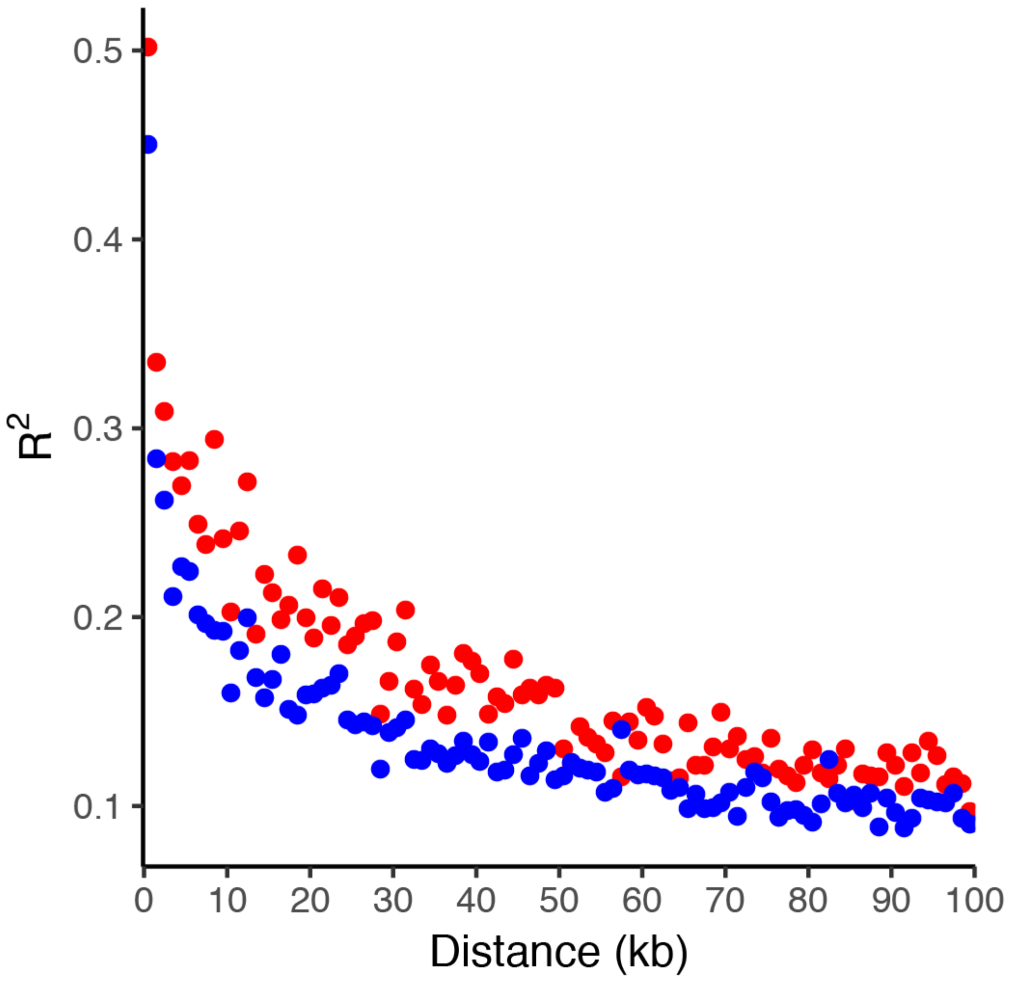

Fig. S10.
Linkage disequilibrium for SNPs found in PAG (red) and GO (blue) samples. Data are binned in 1kb bins. The extent of LD observed in this study are comparable to estimations from *Acropora digitifera* from Ryukyu Archipelago (*80*).

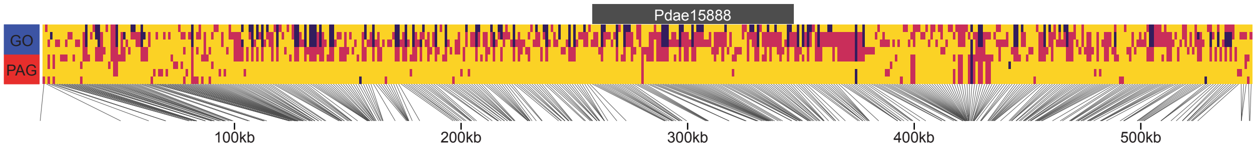

Fig. S11.
Genotypes calls for 481 SNPs along Scaffold 441 derived from bisulfite sequencing. Positions are colored according to genotype call (yellow=homozygous ref; red=heterozygous; blue=homozygous alt). These data from an independent study (*26*) provide additional support for the haplotype structure observed in our study (Fig. 4).

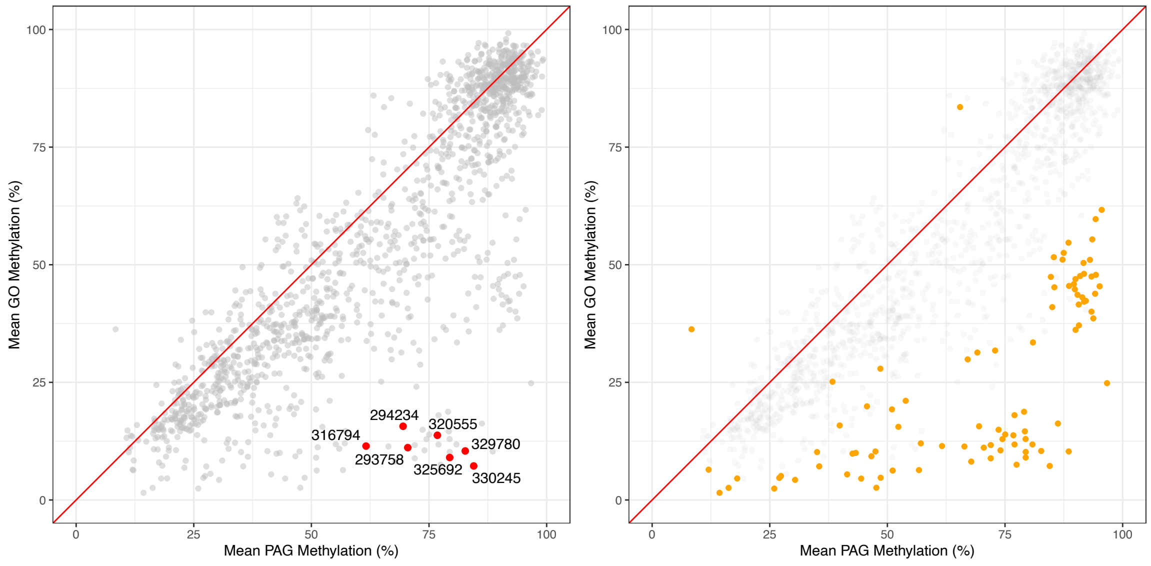

Fig. S12.
Comparison of PAG and GO mean methylation for Pdae15888 methylated sites. Red line indicates a 1:1 ratio. Left panel: Annotated positions in red highlight sites that were selected for Sanger sequencing. Right panel: Annotated positions in orange highlight sites that are putatively mutated in at least one individual.

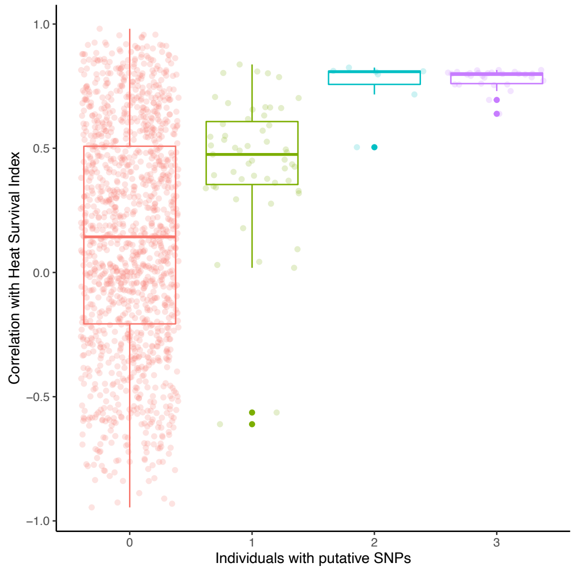

Fig. S13.
Correlation of XRN1 differentially methylated positions with larval heat survival index. Positions categorized based on the number of individuals with methylation patterns supporting the presence of a SNP at that position. The larval heat survival index measures the contribution of sire to larval survival at elevated temperatures with higher scores reflecting better survival. These were calculated from the survival percentage of larvae from multiple experimental crosses exposed to 36°C for 60hrs and were standardized for the effect of dam.

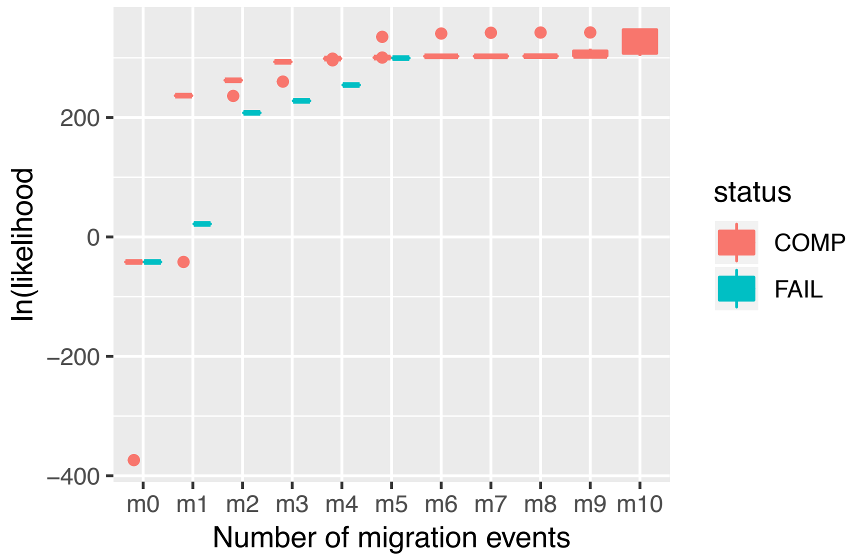

Fig. S14.

Boxplots of the 30 Treemix runs for migration events ranging from zero to ten. The boxplots are coloured according to successful runs (COMP) and those where likelihoods could not be calculated above m=5 (FAIL).

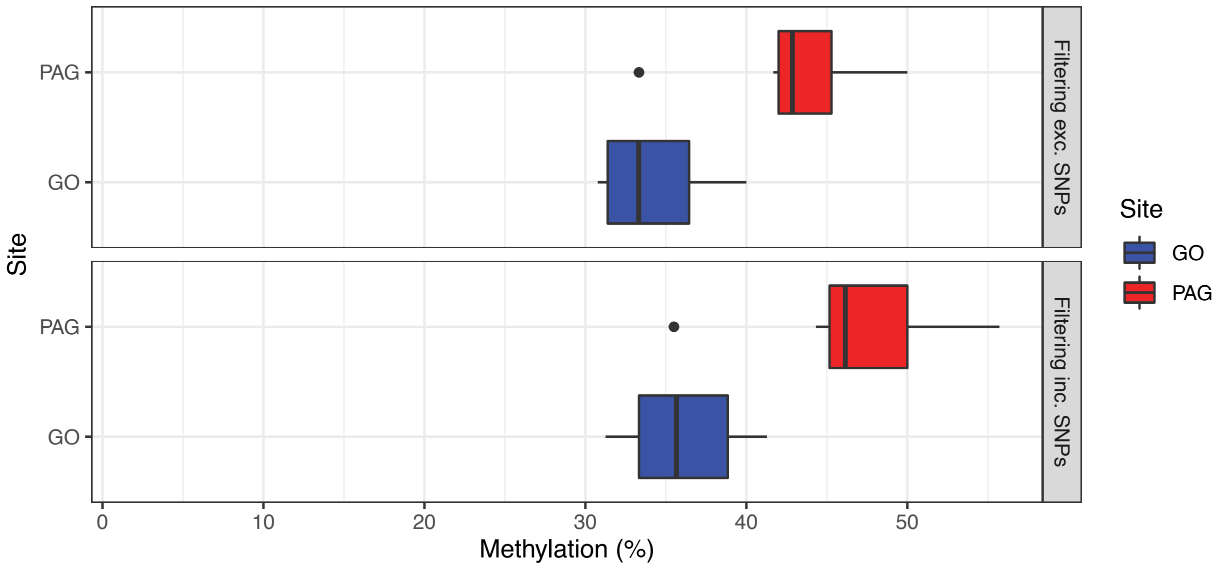

Fig. S15.
Impact of removing putative SNP positions from methylation estimates of gene Pdae15888.

Table S1.

Sampling locations and site abbreviations for this study.

Site Name	Site Abbreviation	Latitude	Longitude
**Qaruh**	KA	28.8333	48.7650
**Umm Al Maradim**	KB	28.6835	48.6557
**Umm Al Arshan**	QB	26.5139	51.2996
**Al Ruwais**	QA	26.2043	51.1784
**Al Ashat**	QC	24.7460	51.6008
**Al Saada**	WC	24.0905	52.1515
**Dhabiya**	DH	24.3655	54.1008
**Ras Ghanada**	RG	24.8482	54.6903
**Sir Bu Nair**	SB	25.2566	54.2095
**Ras al Khaimah**	RK	25.9807	56.0474
**Al Harf**	MA	26.2410	56.1972
**Coral Garden**	MB	26.3769	56.4149
**Dibba Rock**	FD	25.6031	56.3485
**Fahal**	TA	23.6782	58.5010
**Cemetery Bay**	TB	23.6116	58.6038

Table S2.
The proportion of significant three-population tests based on region. Three-population tests were performed for all population combinations. Population A refers to the target population whereas populations B and C are the source populations. For each target population, the source populations are grouped by region (NPAG=northern PAG; SPAG=southern PAG; GO=Gulf of Oman). Each cell reports the number of significant tests (numerator) and the total number of tests (denominator) for each combination of site and source region pair. Using site KA as an example, out of seven tests where the source populations were represented by one SPAG site and one NPAG site, none were significant.

**Pop A**	**Populations B,C**								
	**NPAG, NPAG**	**NPAG, QO**	**NPAG, SPAG**	**NPAG, GO**	**SPAG, SPAG**	**SPAG, QO**	**SPAG, GO**	**GO,GO**	**GO,QO**
**KA**	0/0	0/1	0/7	0/3	0/21	0/7	0/21	0/3	0/3
**KB**	0/0	1/1	7/7	3/3	0/21	0/7	0/21	0/3	0/3
**QB**	0/1	0/0	13/14	6/6	0/21	0/0	12/21	0/3	0/0
**QA**	0/1	0/2	0/12	0/6	0/15	0/6	0/18	0/3	0/3
**QC**	0/1	0/2	0/12	0/6	0/15	0/6	0/18	0/3	0/3
**WC**	0/1	0/2	2/12	0/6	0/15	0/6	0/18	0/3	0/3
**DH**	0/1	0/2	2/12	0/6	0/15	0/6	1/18	0/3	0/3
**RG**	0/1	0/2	0/12	0/6	0/15	0/6	0/18	0/3	0/3
**SB**	0/1	0/2	4/12	0/6	5/15	2/6	18/18	0/3	0/3
**RK**	0/1	0/2	8/12	0/6	5/15	4/6	18/18	0/3	0/3
**MB**	0/1	0/2	0/14	0/4	0/21	0/7	0/14	0/1	0/2
**FD**	0/1	0/2	0/14	0/4	0/21	0/7	0/14	0/1	0/2
**TAB**	0/1	0/2	0/14	0/4	0/21	0/7	0/14	0/1	0/2

Table S3.

Summary statistics for the seven linear models evaluated.
Geo=Geographic distance
Env=Environmental distance
Bar=Presence of barrier at the Strait of Hormuz
K=Number of parameters in the model
AIC=Akaike Information Criterion
RSS=Residual sum of squares

Model	K	AIC	AICc	RSS	R^2	Adj. R^2	Delta.AICc	Model Likelihood	Model Probability	Evidence Ratio
**Geo+Env+Bar**	5	120.58	121.28	17.96	0.80	0.79	0.00	1.000	0.994	1
**Env+Bar**	4	131.03	131.49	20.59	0.77	0.77	10.21	0.006	0.006	165
**Geo+Bar**	4	147.34	147.80	24.64	0.73	0.72	26.52	0.000	0.000	$5.7x10^5$
**Geo+Env**	4	147.72	148.19	24.74	0.73	0.72	26.90	0.000	0.000	$7.0x10^5$
**Env**	3	162.36	162.63	29.70	0.67	0.67	41.35	0.000	0.000	$9.5x10^8$
**Geo**	3	178.97	179.25	35.65	0.60	0.60	57.97	0.000	0.000	$3.9x10^{12}$
**Bar**	3	200.97	201.24	45.40	0.50	0.49	79.96	0.000	0.000	$2.3x10^{17}$

Table S4.
Outlier regions identified on scaffold 27.

Outlier Test	Outlier Region Start Position	Outlier Region End Position
**XPEHH**	1411154	1621714
**Nucleotide Diversity**	1397130	1625802
**F_{ST}**	920751	1049409
	1481656	1625802

Table S5.
Relationship between minor allele frequency and larval survival under elevated temperature for different families of PAG and GO corals. *P-values adjusted using a Benjamini-Hochberg correction.

Index	Tag Start	Tag End	SNP Position	Major Allele	Minor Allele	R^2	Adjusted* P-value
1	1407618	1407653	20	A	G	0.0127	0.6233
2	1429940	1429975	9	C	T	0.1941	0.1313
3	1432646	1432681	29	C	A	0.3209	**0.0269**
4	1513474	1513509	2	T	C	0.0333	0.4396
5	1518935	1518970	11	A	G	0.0532	0.3679
6	1518935	1518970	20	G	A	0.0011	0.8764
7	1519365	1519400	9	A	G	0.1575	0.1313
8	1519365	1519400	12	T	A	0.1575	0.1313
9	1554277	1554312	2	T	C	0.1138	0.2920
10	1565967	1566002	5	A	G	0.1231	0.2050
11	1576513	1576548	29	G	A	0.0818	0.3679
12	1576513	1576548	32	A	G	0.0818	0.3679
13	1576513	1576548	33	G	A	0.0818	0.3679

Table S6.

Effect of putative mutations on the correlation between XRN1 methylation positions and larval heat survival index. P-values report statistical significance based on permutation analysis (10^7 permutations).

Number of individuals with putative SNP in methylated position	Mean Pearson's correlation with larval heat survival index	P-value
0	R = 0.1330	
≥ 1	R = 0.5720	$p < 1x10^{-7}$
≥ 2	R = 0.7754	$p < 1x10^{-7}$
≥ 3	R = 0.7808	$p < 1x10^{-7}$

Table S7.
Allele counts for Sanger sequenced differentially methylated sites in the introns of the XRN1 gene. *[1] CpG denotes the 'intact' CG dinucleotide that, when present, is methylated to some extent. *[2] The Fisher Exact Test was used to test whether the counts of CG vs non-CG dinucleotides differed between Persian/Arabian Gulf and Gulf of Oman samples at each locus.

ID	Differentially methylated position	Alleles (*[1])	PAG Allele Count	GO Allele Count	Fisher Exact Test (*[2])
**P293**	293758	CpG	35	3	*p=1.35e-13*
		CA	5	37	
**P294**	294234	CpG	35	3	*p=1.35e-13*
		CA	5	37	
**P316**	316794	CpG	35	1	*p=7.30e-16*
		CC	5	37	
		CT	0	2	
**P320**	320555	CpG	35	3	*p=1.35e-13*
		TG	5	37	
**P325**	325692	CpG	38	3	*p=1.35e-13*
		CA	2	37	
**P329**	329780	CpG	35	3	*p=1.35e-13*
		CA	5	37	
**P330**	330245	CpG	35	1	*p=1.35e-13*
		CC	5	39	

Table S8.

PCR reaction mixture used in ddRAD library preparation.

Component	Volume (μl)
**Phusion buffer**	6
**$MgCl_2$**	0.75
**dNTPs**	0.6
**PU (Universal primer)**	3
**P2.X**	3
**Phusion enzyme**	0.3
**DNA**	16.35
**TOTAL**	30

Table S9.
Dataset-specific RAD variant filters.

Filter	AseI-BstBI	AseI-MspI
**Minimum depth to call genotype**	5	5
**Minimum mean depth**	10%	-
**Maximum missingness allowed (genotype)**	10%	20%
**Minor allele frequency**	0.05	0.05
**Population specific missingness (genotype)**	20%	25%
**Maximum depth filter**	214	44

Table S10.
Primers used to amplify target CpG sites. The suffix “INT” indicates internal primers used to sequence positions impacted by slippage in original chromatograms.

Primer Name	Sequence 5’-3’	Targeted Position	Location
**293F**	TTGGGCGAGCGACATTAAATGTGC	293758	Intron 1
**294R**	GATGTGCAGTGATATGGCACTAGG	294234	
**316F**	GCTCAAGATTACTGGCTGCATGC	316794	Intron 2
**316R**	GCATTAACCCTATTCGGACGAGTG		
**320F**	TGGGATATTGGAAGTACCATTGCAG	320555	Intron 2
**320R**	CAAGAGTGGAGAGCAGATTGTGC		
**325F**	AAGAACTCATGGAGAGCCAGGTC	325692	Intron 4
**325R**	GCCAAGCAGAATCAAGTACAGTCG		
**329F**	GAAATTCATGCCTTCAGGGTGTTCC	329780	Intron 5
**330R**	CTTTCACTGGACGTTTCCTCTGC	330245	
**294INT**	AATCGGTAATCAGTGGACCAG	293758<br>294234	Intron 1
**330INT**	ACCCAGTTTCCAAGCATGC	329780<br>330245	Intron 5

Table S11.
Methylation differences between PAG and GO corals in genes located in the outlier region identified on Scaffold 27. Data are from Liew *et al.* (*26*) and reanalyzed to include methylation sites that may be subject to mutation.

Gene ID	Persian/Arabian Gulf		Gulf of Oman		Corrected t-test p value
	Mean (%)	SD (%)	Mean (%)	SD (%)	
**Pdae2523**	4.06	7.78	20.09	17.99	0.242
**Pdae2524**	79.85	6.78	82.34	8.17	0.801
**Pdae2529**	88.57	11.40	88.33	6.87	0.987
**Pdae2530**	18.54	13.95	33.65	18.07	0.358

Table S12.
Methylation statistics of gene Pdae15888, a 5-3 exoribonuclease extracted from the dataset of Liew and coworkers (*25*).

	**Filtering exc. putative SNPs**	**Filtering inc. putative SNPs**
**Mean Methylation**	GO: 34.3% PAG: 43.0%	GO: 36.1% PAG: 46.7%
**Absolute Difference (%)**	+8.7% in PAG	+10.6% in PAG
**Relative Difference (%)**	↑25.4% in PAG	↑29.4% in PAG
**Corrected t-test p value**	0.039	0.030

REFERENCES AND NOTES

1. O. Hoegh-Guldberg, J. F. Bruno, The impact of climate change on the world's marine ecosystems. *Science* **328**, 1523–1528 (2010).

2. S. C. Doney, M. Ruckelshaus, J. E. Duffy, J. P. Barry, F. Chan, C. A. English, H. M. Galindo, J. M. Grebmeier, A. B. Hollowed, N. Knowlton, J. Polovina, N. N. Rabalais, W. J. Sydeman, L. D. Talley, Climate change impacts on marine ecosystems. *Annu. Rev. Marine Sci.* **4**, 11–37 (2012).

3. N. Knowlton, R. E. Brainard, R. Fisher, M. Moews, L. Plaisance, M. J. Caley, Coral reef biodiversity, in *Life in the World's Oceans: Diversity Distribution and Abundance* (2010), pp. 65–74.

4. T. P. Hughes, J. T. Kerry, A. H. Baird, S. R. Connolly, A. Dietzel, C. M. Eakin, S. F. Heron, A. S. Hoey, M. O. Hoogenboom, G. Liu, M. J. McWilliam, R. J. Pears, M. S. Pratchett, W. J. Skirving, J. S. Stella, G. Torda, Global warming transforms coral reef assemblages. *Nature* **556**, 492–496 (2018).

5. C. Wilkinson, *Status of Coral Reefs of the World: 2000* (2000).

6. O. Hoegh-Guldberg, Climate change, coral bleaching and the future of the world's coral reefs. *Mar. Freshw. Res.* **50**, 839–866 (1999).

7. P. Craig, C. Birkeland, S. Belliveau, High temperatures tolerated by a diverse assemblage of shallow-water corals in American Samoa. *Coral Reefs* **20**, 185–189 (2001).

8. E. F. Camp, M. R. Nitschke, R. Rodolfo-Metalpa, F. Houlbreque, S. G. Gardner, D. J. Smith, M. Zampighi, D. J. Suggett, Reef-building corals thrive within hot-acidified and deoxygenated waters. *Sci. Rep.* **7**, 2434 (2017).

9. V. Schoepf, M. Stat, J. L. Falter, M. T. McCulloch, Limits to the thermal tolerance of corals adapted to a highly fluctuating, naturally extreme temperature environment. *Sci. Rep.* **5**, 17639 (2015).

10. J. A. Burt, E. F. Camp, I. C. Enochs, J. L. Johansen, K. M. Morgan, B. Riegl, A. S. Hoey, Insights from extreme coral reefs in a changing world. *Coral Reefs* **39**, 495–507 (2020).

11. D. J. Barshis, J. T. Ladner, T. A. Oliver, F. O. Seneca, N. Traylor-Knowles, S. R. Palumbi, Genomic basis for coral resilience to climate change. *Proc. Natl. Acad. Sci.* **110**, 1387–1392 (2013).

12. R. A. Bay, S. R. Palumbi, Multilocus adaptation associated with heat resistance in reef-building corals. *Curr. Biol.* **24**, 2952–2956 (2014).

13. T. A. Oliver, S. R. Palumbi, Many corals host thermally resistant symbionts in high-temperature habitat. *Coral Reefs* **30**, 241–250 (2011).

14. M. Ziegler, F. O. Seneca, L. K. Yum, S. R. Palumbi, C. R. Voolstra, Bacterial community dynamics are linked to patterns of coral heat tolerance. *Nat. Commun.* **8**, 14213 (2017).

15. B. M. Riegl, S. J. Purkis, A. S. Al-Cibahy, M. A. Abdel-Moati, O. Hoegh-Guldberg, Present limits to heat-adaptability in corals and population-level responses to climate extremes. *PLOS ONE* **6**, e24802 (2011).

16. E. G. Smith, G. O. Vaughan, R. N. Ketchum, D. McParland, J. A. Burt, Symbiont community stability through severe coral bleaching in a thermally extreme lagoon. *Sci. Rep.* **7**, 2428 (2017).

17. J. A. Burt, F. Paparella, N. Al-Mansoori, A. Al-Mansoori, H. Al-Jailani, Causes and consequences of the 2017 coral bleaching event in the southern Persian/Arabian Gulf. *Coral Reefs* **38**, 567–589 (2019).

18. E. J. Howells, D. Abrego, E. Meyer, N. L. Kirk, J. A. Burt, Host adaptation and unexpected symbiont partners enable reef-building corals to tolerate extreme temperatures. *Glob. Chang. Biol.* **22**, 2702–2714 (2016).

19. B. Hume, C. D'Angelo, J. Burt, A.C. Baker, B. Riegl, J. Wiedenmann, Corals from the Persian/Arabian Gulf as models for thermotolerant reef-builders: Prevalence of clade C3 Symbiodinium, host fluorescence and ex situ temperature tolerance. *Mar. Pollut. Bull.* **72**, 313–322 (2013).

20. E. J. Howells, A. G. Bauman, G. O. Vaughan, B. C. C. Hume, C. R. Voolstra, J. A. Burt, Corals in the hottest reefs in the world exhibit symbiont fidelity not flexibility. *Mol. Ecol.* **29**, 899–911 (2020).

21. B. C. Hume, C. D'Angelo, E. G. Smith, J. R. Stevens, J. Burt, J. Wiedenmann, *Symbiodinium thermophilum* sp. nov., a thermotolerant symbiotic alga prevalent in corals of the world's hottest sea, the Persian/Arabian Gulf. *Sci. Rep.* **5**, 8562 (2015).

22. B. C. Hume, C. R. Voolstra, C. Arif, C. D'Angelo, J. A. Burt, G. Eyal, Y. Loya, J. Wiedenmann, Ancestral genetic diversity associated with the rapid spread of stress-tolerant coral symbionts in response to Holocene climate change. *Proc. Natl. Acad. Sci. U.S.A.* **113**, 4416–4421 (2016).

23. E. G. Smith, B. C. Hume, P. Delaney, J. Wiedenmann, J. A. Burt, Genetic structure of coral-Symbiodinium symbioses on the world's warmest reefs. *PLOS ONE* **12**, e0180169 (2017).

24. E. G. Smith, R. N. Ketchum, J. A. Burt, Host specificity of Symbiodinium variants revealed by an ITS2 metahaplotype approach. *ISME J.* **11**, 1500–1503 (2017).

25. N. L. Kirk, E. J. Howells, D. Abrego, J. A. Burt, E. Meyer, Genomic and transcriptomic signals of thermal tolerance in heat-tolerant corals (Platygyra daedalea) of the Arabian/Persian Gulf. *Mol. Ecol.* **27**, 5180–5194 (2018).

26. Y. J. Liew, E. J. Howells, X. Wang, C. T. Michell, J. A. Burt, Y. Idaghdour, M. Aranda, Intergenerational epigenetic inheritance in reef-building corals. *Nat. Clim. Chang.* **10**, 254–259 (2020).

27. K. Lambeck, Shoreline reconstructions for the Persian Gulf since the last glacial maximum. *Earth Planet. Sci. Lett.* **142**, 43–57 (1996).

28. C. M. Eakin, H. P. Sweatman, R. E. Brainard, The 2014–2017 global-scale coral bleaching event: Insights and impacts. *Coral Reefs* **38**, 539–545 (2019).

29. J. Burt, S. Al-Harthi, A. Al-Cibahy, Long-term impacts of coral bleaching events on the world's warmest reefs. *Mar. Environ. Res.* **72**, 225–229 (2011).

30. J. A. Burt, E. G. Smith, C. Warren, J. Dupont, An assessment of Qatar's coral communities in a regional context. *Mar. Pollut. Bull.* **105**, 473–479 (2016).

31. J. P. Hoolihan, J. Premanandh, M.-A. D'Aloia-Palmieri, J. A. Benzie, Intraspecific phylogeographic isolation of Arabian Gulf sailfish Istiophorus platypterus inferred from mitochondrial DNA. *Mar. Biol.* **145**, 465–475 (2004).

32. B. Riegl, Climate change and coral reefs: Different effects in two high-latitude areas (Arabian Gulf, South Africa). *Coral reefs* **22**, 433–446 (2003).

33. J. A. Burt, The environmental costs of coastal urbanization in the Arabian Gulf. *City* **18**, 760–770 (2014).

34. B. Riegl, M. Johnston, S. Purkis, E. Howells, J. Burt, S. C. C. Steiner, C. R. C. Sheppard, A. Bauman, Population collapse dynamics in Acropora downingi, an Arabian/Persian Gulf ecosystem-engineering coral, linked to rising temperature. *Glob. Chang. Biol.* **24**, 2447–2462 (2018).

35. J. K. Pritchard, M. Stephens, P. Donnelly, Inference of population structure using multilocus genotype data. *Genetics* **155**, 945–959 (2000).

36. I. J. Wang, G. S. Bradburd, Isolation by environment. *Mol. Ecol.* **23**, 5649–5662 (2014).

37. D. Marshall, K. Monro, M. Bode, M. Keough, S. Swearer, Phenotype–environment mismatches reduce connectivity in the sea. *Ecol. Lett.* **13**, 128–140 (2010).

38. P. Saenz-Agudelo, J. D. Dibattista, M. J. Piatek, M. R. Gaither, H. B. Harrison, G. B. Nanninga, M. L. Berumen, Seascape genetics along environmental gradients in the Arabian Peninsula: Insights from ddRAD sequencing of anemonefishes. *Mol. Ecol.* **24**, 6241–6255 (2015).

39. C. Sheppard, M. al-Husiani, F. al-Jamali, F. al-Yamani, R. Baldwin, J. Bishop, F. Benzoni, E. Dutrieux, N. K. Dulvy, S. R. V. Durvasula, D. A. Jones, R. Loughland, D. Medio, M. Nithyanandan, G. M. Pilling, I. Polikarpov, A. R.G. Price, S. Purkis, B. Riegl, M. Saburova, K. S. Namin, O. Taylor, S. Wilson, K. Zainal, The Gulf: A young sea in decline. *Mar. Pollut. Bull.* **60**, 13–38 (2010).

40. J. K. Pickrell, J. K. Pritchard, Inference of population splits and mixtures from genome-wide allele frequency data. *PLOS Genet.* **8**, e1002967 (2012).

41. R. M. Reynolds, Physical oceanography of the Gulf, Strait of Hormuz, and the Gulf of Oman—Results from the *Mt Mitchell* expedition. *Mar. Pollut. Bull.* **27**, 35–59 (1993).

42. X. Liu, Y.-X. Fu, Exploring population size changes using SNP frequency spectra. *Nat. Genet.* **47**, 555–559 (2015).

43. M. Schubert, H. Jónsson, D. Chang, C. der Sarkissian, L. Ermini, A. Ginolhac, A. Albrechtsen, I. Dupanloup, A. Foucal, B. Petersen, M. Fumagalli, M. Raghavan, A. Seguin-Orlando, T. S. Korneliussen, A. M. V. Velazquez, J. Stenderup, C. A. Hoover, C.J. Rubin, A. H. Alfarhan, S. A. Alquraishi, K. A. S. al-Rasheid, D. E. MacHugh, T. Kalbfleisch, J. N. MacLeod, E. M. Rubin, T. Sicheritz-Ponten, L. Andersson, M. Hofreiter, T. Marques-Bonet, M. T. P. Gilbert, R. Nielsen, L. Excoffier, E. Willerslev, B. Shapiro, L. Orlando, Prehistoric genomes reveal the genetic foundation and cost of horse domestication. *Proc. Natl. Acad. Sci.* **111**, E5661–E5669 (2014).

44. R. N. Ketchum, E. G. Smith, M. B. DeBiasse, G. O. Vaughan, D. McParland, W. B. Leach, N. Al-Mansoori, J. F. Ryan, J. A. Burt, A. M. Reitzel, Population genomic analyses of the sea urchin *Echinometra* sp. EZ across an extreme environmental gradient. *Genome Biol. Evol.* **12**, 1819–1829 (2020)

45. S. J. Purkis, D. A. Renegar, B. M. Riegl, The most temperature-adapted corals have an Achilles' Heel. *Mar. Pollut. Bull.* **62**, 246–250 (2011).

46. G. B. Dixon, S. W. Davies, G. V. Aglyamova, E. Meyer, L. K. Bay, M. V. Matz, Genomic determinants of coral heat tolerance across latitudes. *Science* **348**, 1460–1462 (2015).

47. J. A. Kleypas, D. M. Thompson, F. S. Castruccio, E. N. Curchitser, M. Pinsky, J. R. Watson, Larval connectivity across temperature gradients and its potential effect on heat tolerance in coral populations. *Glob. Chang. Biol.* **22**, 3539–3549 (2016).

48. H. M. Putnam, K. L. Barott, T. D. Ainsworth, R. D. Gates, The vulnerability and resilience of reef-building corals. *Curr. Biol.* **27**, R528–R540 (2017).

49. M. V. Matz, E. A. Treml, G. V. Aglyamova, L. K. Bay, Potential and limits for rapid genetic adaptation to warming in a Great Barrier Reef coral. *PLOS Genet.* **14**, e1007220 (2018).

50. E. J. Howells, D. Abrego, Y. J. Liew, J. A. Burt, M. Aranda, Enhancing the heat tolerance of reef-building corals to future warming. *Sci. Adv.* **7**, eabg6070 (2021).

51. M. K. DeSalvo, A. Estrada, S. Sunagawa, M. Medina, Transcriptomic responses to darkness stress point to common coral bleaching mechanisms. *Coral Reefs* **31**, 215–228 (2012).

52. M. K. DeSalvo, S. Sunagawa, C. R. Voolstra, M. Medina, Transcriptomic responses to heat stress and bleaching in the elkhorn coral Acropora palmata. *Mar. Ecol. Prog. Ser.* **402**, 97–113 (2010).

53. M. Ricaurte, N. V. Schizas, P. Ciborowski, N. M. Boukli, Proteomic analysis of bleached and unbleached Acropora palmata, a threatened coral species of the Caribbean. *Mar. Pollut. Bull.* **107**, 224–232 (2016).

54. L. J. Ruiz-Jones, S. R. Palumbi, Tidal heat pulses on a reef trigger a fine-tuned transcriptional response in corals to maintain homeostasis. *Sci. Adv.* **3**, e1601298 (2017).

55. M. E. Strader, G. V. Aglyamova, M. V. Matz, Red fluorescence in coral larvae is associated with a diapause-like state. *Mol. Ecol.* **25**, 559–569 (2016).

56. M. DeSalvo, C. R. Voolstra, S. Sunagawa, J. A. Schwarz, J. H. Stillman, M. A. Coffroth, A. M. Szmant, M. Medina, Differential gene expression during thermal stress and bleaching in the Caribbean coral Montastraea faveolata. *Mol. Ecol.* **17**, 3952–3971 (2008).

57. A. J. Weston, W. C. Dunlap, V. H. Beltran, A. Starcevic, D. Hranueli, M. Ward, P. F. Long, Proteomics links the redox state to calcium signaling during bleaching of the scleractinian coral Acropora microphthalma on exposure to high solar irradiance and thermal stress. *Mol. Cell. Proteomics* **14**, 585–595 (2015).

58. P. A. Cleves, C. J. Krediet, E. M. Lehnert, M. Onishi, J. R. Pringle, Insights into coral bleaching under heat stress from analysis of gene expression in a sea anemone model system. *Proc. Natl. Acad. Sci.* **117**, 28906–28917 (2020).

59. P. A. Cleves, A. I. Tinoco, J. Bradford, D. Perrin, L. K. Bay, J. R. Pringle, Reduced thermal tolerance in a coral carrying CRISPR-induced mutations in the gene for a heat-shock transcription factor. *Proc. Natl. Acad. Sci.* **117**, 28899–28905 (2020).

60. Y. Li, Y. J. Liew, G. Cui, M. J. Cziesielski, N. Zahran, C. T. Michell, C. R. Voolstra, M. Aranda, DNA methylation regulates transcriptional homeostasis of algal endosymbiosis in the coral model Aiptasia. *Sci. Adv.* **4**, eaat2142 (2018).

61. G. Dixon, Y. Liao, L. K. Bay, M. V. Matz, Role of gene body methylation in acclimatization and adaptation in a basal metazoan. *Proc. Natl. Acad. Sci.* **115**, 13342–13346 (2018).

62. A. H. Nguyen, A. Matsui, M. Tanaka, K. Mizunashi, K. Nakaminami, M. Hayashi, K. Iida, T. Toyoda, D. V. Nguyen, M. Seki, Loss of Arabidopsis 5′–3′ exoribonuclease AtXRN4 function enhances heat stress tolerance of plants subjected to severe heat stress. *Plant Cell Physiol.* **56**, 1762–1772 (2015).

63. R. Merret, J. Descombin, Y. T. Juan, J. J. Favory, M. C. Carpentier, C. Chaparro, Y. Y. Charng, J. M. Deragon, C. Bousquet-Antonelli, XRN4 and LARP1 are required for a heat-triggered mRNA decay pathway involved in plant acclimation and survival during thermal stress. *Cell Rep.* **5**, 1279–1293 (2013).

64. W. R. Rideout, G. A. Coetzee, A. F. Olumi, P. A. Jones, 5-Methylcytosine as an endogenous mutagen in the human LDL receptor and p53 genes. *Science* **249**, 1288–1290 (1990).

65. D. Anastasiadi, A. Esteve-Codina, F. Piferrer, Consistent inverse correlation between DNA methylation of the first intron and gene expression across tissues and species. *Epigenetics Chromatin* **11**, 37 (2018).

66. X. Zhang, M. Wu, H. Xiao, M. T. Lee, L. Levin, Y. K. Leung, S. M. Ho, Methylation of a single intronic CpG mediates expression silencing of the PMP24 gene in prostate cancer. *Prostate* **70**, 765–776 (2010).

67. A. Blattler, L. Yao, H. Witt, Y. Guo, C. M. Nicolet, B. P. Berman, P. J. Farnham, Global loss of DNA methylation uncovers intronic enhancers in genes showing expression changes. *Genome Biol.* **15**, 469 (2014).

68. B. K. Peterson, J. N. Weber, E. H. Kay, H. S. Fisher, H. E. Hoekstra, Double digest RADseq: An inexpensive method for de novo SNP discovery and genotyping in model and non-model species. *PLOS ONE* **7**, e37135 (2012).

69. J. B. Puritz, C. M. Hollenbeck, J. R. Gold, dDocent: A RADseq, variant-calling pipeline designed for population genomics of non-model organisms. *PeerJ* **2**, e431 (2014).

70. J. Catchen, P. A. Hohenlohe, S. Bassham, A. Amores, W. A. Cresko, Stacks: An analysis tool set for population genomics. *Mol. Ecol.* **22**, 3124–3140 (2013).

71. C. C. Chang, C. C. Chow, L. C. Tellier, S. Vattikuti, S. M. Purcell, J. J. Lee, Second-generation PLINK: Rising to the challenge of larger and richer datasets. *Gigascience* **4**, 7 (2015).

72. M. Jakobsson, N. A. Rosenberg, CLUMPP: A cluster matching and permutation program for dealing with label switching and multimodality in analysis of population structure. *Bioinformatics* **23**, 1801–1806 (2007).

73. P. A. Hohenlohe, S. Bassham, P. D. Etter, N. Stiffler, E. A. Johnson, W. A. Cresko, Population genomics of parallel adaptation in threespine stickleback using sequenced RAD tags. *PLOS Genet.* **6**, e1000862 (2010).

74. K. M. Hazzouri, J. M. Flowers, H. J. Visser, H. S. M. Khierallah, U. Rosas, G. M. Pham, R. S. Meyer, C. K. Johansen, Z. A. Fresquez, K. Masmoudi, N. Haider, N. el Kadri, Y. Idaghdour, J. A. Malek, D. Thirkhill, G. S. Markhand, R. R. Krueger, A. Zaid, M. D. Purugganan, Whole genome re-sequencing of date palms yields insights into diversification of a fruit tree crop. *Nat. Commun.* **6**, 8824 (2015).

75. P. C. Sabeti, P. Varilly, B. Fry, J. Lohmueller, E. Hostetter, C. Cotsapas, X. Xie, E. H. Byrne, S. McCarroll, R. Gaudet, S. F. Schaffner, E. S. Lander; International HapMap Consortium, K. A. Frazer, D. G. Ballinger, D. R. Cox, D. A. Hinds, L. L. Stuve, R. A. Gibbs, J. W. Belmont, A.

Boudreau, P. Hardenbol, S. M. Leal, S. Pasternak, D. A. Wheeler, T. D. Willis, F. Yu, H. Yang, C. Zeng, Y. Gao, H. Hu, W. Hu, C. Li, W. Lin, S. Liu, H. Pan, X. Tang, J. Wang, W. Wang, J. Yu, B. Zhang, Q. Zhang, H. Zhao, H. Zhao, J. Zhou, S. B. Gabriel, R. Barry, B. Blumenstiel, A. Camargo, M. Defelice, M. Faggart, M. Goyette, S. Gupta, J. Moore, H. Nguyen, R. C. Onofrio, M. Parkin, J. Roy, E. Stahl, E. Winchester, L. Ziaugra, D. Altshuler, Y. Shen, Z. Yao, W. Huang, X. Chu, Y. He, L. Jin, Y. Liu, Y. Shen, W. Sun, H. Wang, Y. Wang, Y. Wang, X. Xiong, L. Xu, M. M. Waye, S. K. Tsui, H. Xue, J. T. Wong, L. M. Galver, J. B. Fan, K. Gunderson, S. S. Murray, A. R. Oliphant, M. S. Chee, A. Montpetit, F. Chagnon, V. Ferretti, M. Leboeuf, J. F. Olivier, M. S. Phillips, S. Roumy, C. Sallée, A. Verner, T. J. Hudson, P. Y. Kwok, D. Cai, D. C. Koboldt, R. D. Miller, L. Pawlikowska, P. Taillon-Miller, M. Xiao, L. C. Tsui, W. Mak, Y. Q. Song, P. K. Tam, Y. Nakamura, T. Kawaguchi, T. Kitamoto, T. Morizono, A. Nagashima, Y. Ohnishi, A. Sekine, T. Tanaka, T. Tsunoda, P. Deloukas, C. P. Bird, M. Delgado, E. T. Dermitzakis, R. Gwilliam, S. Hunt, J. Morrison, D. Powell, B. E. Stranger, P. Whittaker, D. R. Bentley, M. J. Daly, P. I. de Bakker, J. Barrett, Y. R. Chretien, J. Maller, S. McCarroll, N. Patterson, I. Pe'er, A. Price, S. Purcell, D. J. Richter, P. Sabeti, R. Saxena, S. F. Schaffner, P. C. Sham, P. Varilly, D. Altshuler, L. D. Stein, L. Krishnan, A. V. Smith, M. K. Tello-Ruiz, G. A. Thorisson, A. Chakravarti, P. E. Chen, D. J. Cutler, C. S. Kashuk, S. Lin, G. R. Abecasis, W. Guan, Y. Li, H. M. Munro, Z. S. Qin, D. J. Thomas, G. McVean, A. Auton, L. Bottolo, N. Cardin, S. Eyheramendy, C. Freeman, J. Marchini, S. Myers, C. Spencer, M. Stephens, P. Donnelly, L. R. Cardon, G. Clarke, D. M. Evans, A. P. Morris, B. S. Weir, T. Tsunoda, T. A. Johnson, J. C. Mullikin, S. T. Sherry, M. Feolo, A. Skol, H. Zhang, C. Zeng, H. Zhao, I. Matsuda, Y. Fukushima, D. R. Macer, E. Suda, C. N. Rotimi, C. A. Adebamowo, I. Ajayi, T. Aniagwu, P. A. Marshall, C. Nkwodimmah, C. D. Royal, M. F. Leppert, M. Dixon, A. Peiffer, R. Qiu, A. Kent, K. Kato, N. Niikawa, I. F. Adewole, B. M. Knoppers, M. W. Foster, E. W. Clayton, J. Watkin, R. A. Gibbs, J. W. Belmont, D. Muzny, L. Nazareth, E. Sodergren, G.M. Weinstock, D. A. Wheeler, I. Yakub, S. B. Gabriel, R. C. Onofrio, D. J. Richter, L. Ziaugra, B. W. Birren, M. J. Daly, D. Altshuler, R. K. Wilson, L. L. Fulton, J. Rogers, J. Burton, N. P. Carter, C. M. Clee, M. Griffiths, M. C. Jones, K. McLay, R. W. Plumb, M. T. Ross, S. K. Sims, D. L. Willey, Z. Chen, H. Han, L. Kang, M. Godbout, J. C. Wallenburg, P. L'Archevêque, G. Bellemare, K. Saeki, H. Wang, D. An, H. Fu, Q. Li, Z. Wang, R. Wang, A. L. Holden, L. D. Brooks, J. McEwen, M. S. Guyer, V. O. Wang, J. L. Peterson, M. Shi, J. Spiegel, L. M. Sung, L. F. Zacharia, F. S. Collins, K. Kennedy, R. Jamieson,

J. Stewart, Genome-wide detection and characterization of positive selection in human populations. *Nature* **449**, 913–918 (2007).

76. S. R. Browning, B. L. Browning, Rapid and accurate haplotype phasing and missing-data inference for whole-genome association studies by use of localized haplotype clustering. *Am. J. Hum. Genet.* **81**, 1084–1097 (2007).

77. Z. A. Szpiech, R. D. Hernandez, selscan: An efficient multithreaded program to perform EHH-based scans for positive selection. *Mol. Biol. Evol.* **31**, 2824–2827 (2014).

78. M. Gautier, R. Vitalis, rehh: An R package to detect footprints of selection in genome-wide SNP data from haplotype structure. *Bioinformatics* **28**, 1176–1177 (2012).

79. J. W. Leigh, D. Bryant, POPART: Full-feature software for haplotype network construction. *Methods Ecol. Evol.* **6**, 1110–1116 (2015).

80. C. Shinzato, S. Mungpakdee, N. Arakaki, N. Satoh, Genome-wide SNP analysis explains coral diversity and recovery in the Ryukyu Archipelago. *Sci. Rep.* **5**, 18211 (2016).

81. T. S. Korneliussen, A. Albrechtsen, R. Nielsen, ANGSD: Analysis of next generation sequencing data. *BMC Bioinformatics* **15**, 356 (2014).

82. H. Li, Aligning sequence reads, clone sequences and assembly contigs with BWA-MEM (2013); arXiv:1303.3997.

83. C. Prada, M. B. DeBiasse, J. E. Neigel, B. Yednock, J. L. Stake, Z. H. Forsman, I. B. Baums, M. E. Hellberg, Genetic species delineation among branching Caribbean Porites corals. *Coral Reefs* **33**, 1019–1030 (2014).

84. C. Prada, B. Hanna, A. F. Budd, C. M. Woodley, J. Schmutz, J. Grimwood, R. Iglesias-Prieto, J. M. Pandolfi, D. Levitan, K. G. Johnson, N. Knowlton, H. Kitano, M. DeGiorgio, M. Medina, Empty niches after extinctions increase population sizes of modern corals. *Curr. Biol.* **26**, 3190–3194 (2016).

85. R. C. Babcock, Comparative demography of three species of scleractinian corals using Age- and size-dependent classifications. *Ecol. Monographs* **61**, 225–244 (1991).

86. J. A. Kleypas, J. W. McManus, L. A. Menez, Environmental limits to coral reef development: Where do we draw the line? *Am. Zool.* **39**, 146–159 (1999).

87. P. Danecek, A. Auton, G. Abecasis, C. A. Albers, E. Banks, M. A. DePristo, R. E. Handsaker, G. Lunter, G. T. Marth, S. T. Sherry, G. McVean, R. Durbin; 1000 Genomes Project Analysis Group, The variant call format and VCFtools. *Bioinformatics* **27**, 2156–2158 (2011).

88. Y. J. Liew, D. Zoccola, Y. Li, E. Tambutté, A. A. Venn, C. T. Michell, G. Cui, E. S. Deutekom, J. A. Kaandorp, C. R. Voolstra, S. Forêt, D. Allemand, S. Tambutté, M. Aranda, Epigenome-associated phenotypic acclimatization to ocean acidification in a reef-building coral. *Sci. Adv.* **4**, eaar8028 (2018).
